# New emerging materials with potential antibacterial activities

**DOI:** 10.1007/s00253-024-13337-6

**Published:** 2024-11-14

**Authors:** Hadeer M. Bedair, Mahmoud Hamed, Fotouh R. Mansour

**Affiliations:** 1 Department of Microbiology and Immunology, Faculty of Pharmacy, Misr University for Science and Technology, 6Th of October City, Egypt; 2 Pharmaceutical Chemistry Department, Faculty of Pharmacy, Misr International University, Km 28 Ismailia Road, Cairo, 44971 Egypt; 3 MIU Chemistry Society (MIU-CS), Faculty of Pharmacy, Misr International University, Km 28 Ismailia Road, Cairo, 44971 Egypt; 4 Pharmaceutical Analytical Chemistry Department, Faculty of Pharmacy, The Medical Campus of Tanta University, Elgeish Street, Tanta, 31111 Egypt

**Keywords:** Antibacterial resistance, Metal–organic frameworks, Covalent organic frameworks, Carbon quantum dots, Ionic liquids, Antibiotics

## Abstract

**Abstract:**

The increasing prevalence of multidrug-resistant pathogens is a critical public health issue, necessitating the development of alternative antibacterial agents. Examples of these pathogens are methicillin-resistant *Staphylococcus aureus* (MRSA) and the emergence of “pan-resistant” Gram-negative strains, such as *Pseudomonas aeruginosa* and *Acinetobacter baumannii*, which occurred more recently. This review examines various emerging materials with significant antibacterial activities. Among these are nanomaterials such as quantum dots, carbon quantum dots, metal–organic frameworks (MOFs), covalent organic frameworks (COFs), and layered double hydroxides, all of which demonstrate excellent antibacterial properties. Interestingly, including antibacterial agents within the structure of these materials can help avoid bacterial resistance and improve the long-term efficacy of the materials. Additionally, the antibacterial potential of liquid solvents, including ionic liquids and both deep eutectic solvents and natural deep eutectic solvents, is explored. The review discusses the synthesis methods, advantages, and antibacterial efficacy of these new materials. By providing a comprehensive overview of these innovative materials, this review aims to contribute to the ongoing search for effective solutions to combat antibiotic resistance. Key studies demonstrating antibacterial effects against pathogens like *Escherichia coli*, *Staphylococcus aureus*, and multidrug-resistant strains are summarized. MOFs have exhibited antibacterial properties through controlled ion release and surface interactions. COFs have enhanced the efficacy of encapsulated antibiotics and displayed intrinsic antibacterial activity. Other nanomaterials, such as quantum dots, have generated reactive oxygen species, leading to microbial inactivation. This review aims to provide insights into these new classes of antibacterial materials and highlight them for addressing the global crisis of antibiotic resistance.

**Key points:**

*• Nanomaterials show strong antibacterial effects against drug-resistant bacteria*

*• Emerging solvents like ionic liquids offer novel solutions for bacterial resistance*

*• MOFs and COFs enhance antibiotic efficacy, showing promise in combating resistance*

**Supplementary Information:**

The online version contains supplementary material available at 10.1007/s00253-024-13337-6.

## Introduction

Antibacterials play a crucial role in combating infections caused by pathogenic bacteria. These agents are essential in both medical and non-medical settings to prevent and treat various infectious diseases. The effectiveness of antibacterials lies in their ability to inhibit the growth of or kill these pathogens, thereby helping to control and manage infectious diseases (Di Martino 2022). Antibacterial agents have been used for more than 75 years, but these drugs remain a significant issue. Currently, there is a significant global concern since antibacterials are becoming less efficient in combating bacteria, resulting in a decline in their capacity to treat infections (Hochvaldová et al. 2022). According to estimates, if resistance to antibiotics continues to rise at its current pace, untreatable illnesses caused by multidrug-resistant bacteria would surpass all other causes of mortality by 2050 (Nikfarjam et al. 2021). The growing resistance to antibacterial treatments presents the potential for a regression to the pre-antibiotic period, whereby effective medications will be inaccessible for the treatment of bacterial diseases caused by multidrug-resistant bacteria.

The indiscriminate and unnecessary utilization of antibiotics over the past 50 years has significantly contributed to the rapid development of these highly resistant microorganisms (Nikfarjam et al. 2021). According to the World Health Organization (WHO), a minimum of 700,000 individuals pass annually as a result of these bacterial illnesses. In addition, in nations experiencing extreme poverty, antibiotic-resistant bacterial illnesses may result in approximately 24 million deaths every year. An instance of completely antibiotic-resistant TB in India, Iran, and Italy is linked to the ability of disease-causing bacteria to adapt to several drugs (CDC 2019; Nikfarjam et al. 2021). A number of variables lead to this problem: a significant portion of infections caused by bacteria originate from the human microflora, there is a growing resistance to antibacterial drugs, and the frequent use of invasive diagnostic and therapeutic procedures also contributes to the rise in antibacterial drug resistance.

An inherent challenge in addressing bacterial resistance is the relentless dissemination of antibiotic resistance, which persists irrespective of antibiotic usage. The reason for this is the rise and spread of resistance through a technique known as genetic transfer, whereby genetic information is passed from bacteria that are resistant to those that are not resistant through recombination processes (Report 2014). The emergence and dissemination of bacterial resistance are seen as inherent phenomena that cannot be entirely avoided but may be impacted in both beneficial and detrimental ways (Ahmed et al. 2024). The majority of resistance mechanisms in bacteria evolved prior to the introduction of contemporary antibacterial agents, mostly due to the fact that many antibacterial drugs are derived from substances generated by other microbes. Bacterial resistance mechanisms do not usually arise randomly, but instead, they remain dormant until favorable conditions arise for their growth and spread across bacterial populations (Ahmed et al. 2024). The emergence of antibiotic resistance in *Staphylococcus aureus* against β-lactam antibiotics has been a major cause for concerns. After the advent of penicillin for therapy, there was a quick rise in the number of strains that were resistant to penicillin. During the early 1940s, the proportion of *Staphylococcus aureus* strains in English hospitals that were resistant to penicillin was under 1%. However, by 1946, this percentage had increased dramatically to 60%. Patients afflicted with multidrug-resistant bacteria have exhibited elevated death rates in comparison to those infected with susceptible strains of the identical species (Ahmed et al. 2024). Herkel et al. (2016) found a notable difference in death rates for ventilator-associated pneumonia between patients who received appropriate antibiotic therapy (27% mortality rate) and those who received insufficient medication (45% mortality rate). However, more than 50% of antibiotic drug prescriptions in ICUs are for ventilator-associated pneumonia, highly contributing to the emergence of antibacterial-resistant pathogens worldwide (Miron et al. 2024).

**Fig. 1:**
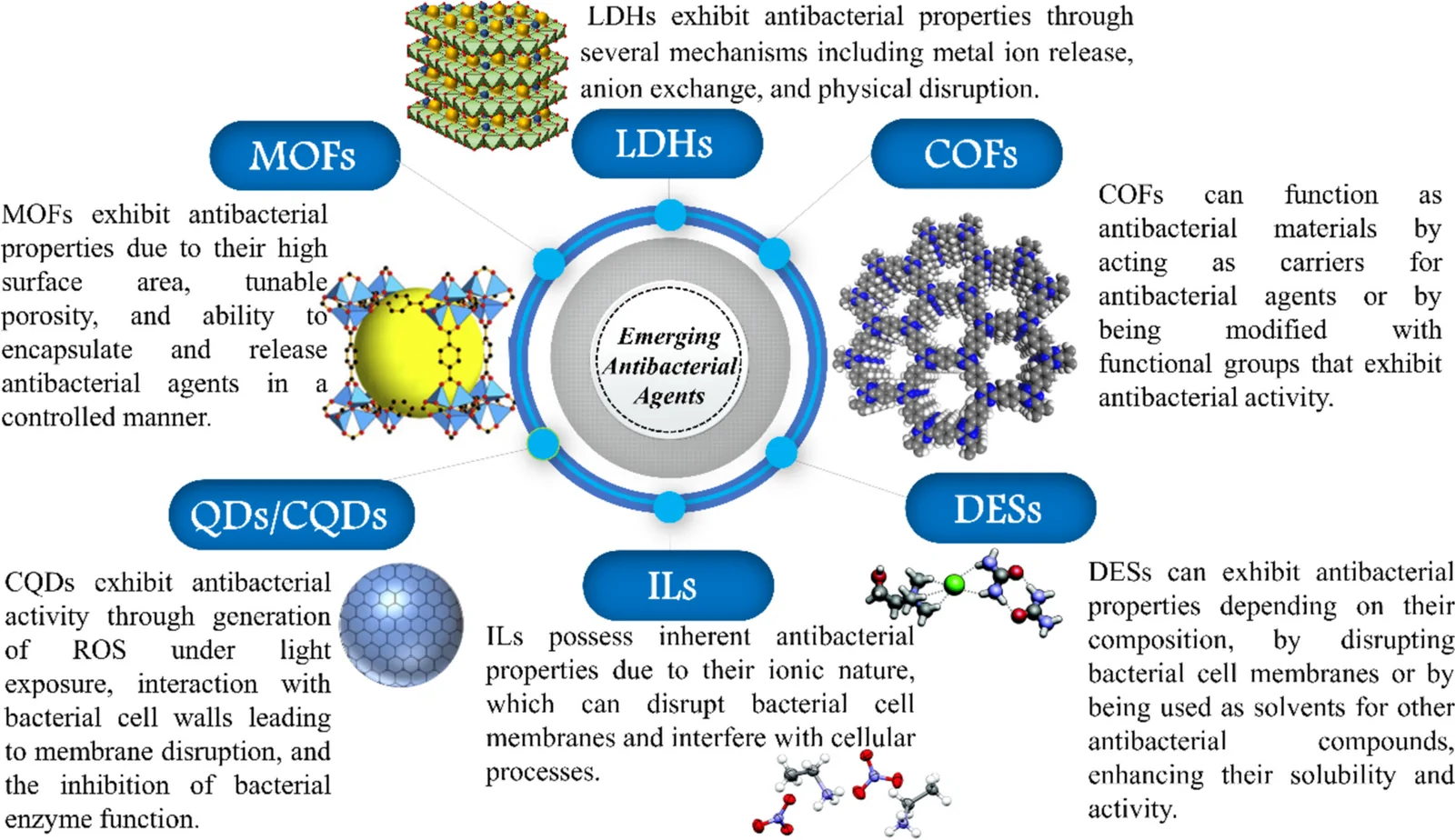
Main classes of new emerging materials with antibacterial activities

The inevitability of bacterial resistance persists despite preventive measures, with nanotechnology emerging as a promising solution. Combining existing antibacterials with silver nanoparticles has shown high efficacy against various bacteria, including multidrug-resistant strains like methicillin-resistant *Staphylococcus aureus* and vancomycin-resistant *enterococci* (Hochvaldová et al. 2022). The urgent global goal in the post-antibiotic era is the development of novel antibacterial materials, with metal–organic frameworks (MOFs) (Chen et al. 2020), covalent organic frameworks (COFs) (Gendy et al. 2022), quantum dots (QDs) (Tian et al. 2019), carbon quantum dots (CQDs) (Travlou et al. 2018), ionic liquids (ILs) (Gao et al. 2024), layered double hydroxide (LDH) (Tang et al. 2018), and deep eutectic solvents (DES) (Wojeicchowski et al. 2021) being promising candidates with antibacterial properties against diverse pathogens. Since their establishment, nanoparticles and nanoarchitectures have transformed the area of materials science by providing a multitude of unique qualities such as high surface-to-volume ratios and specialized size- and surface-dependent characteristics (Al-dolaimy et al. 2024). These nanostructures include the previously listed materials. An example of why these materials represent a significant advance over traditional antibiotics is that incorporating antibacterial agents within the COF structure can help prevent bacterial resistance and improve the COF’s long-term efficacy (Al-dolaimy et al. 2024). As a result, COFs can be engineered to optimize their antibacterial effectiveness while minimizing toxicity, making them a promising alternative to standard antibiotics for the treatment of bacterial infections (Al-dolaimy et al. 2024). Figure 1 summarizes the main classes of materials with emerging antibacterial activities. This review will delve into each class, detailing their preparation methods and advantages and showcasing their antibacterial activities against various microorganisms.

## New materials as antibacterial agents

### Metal–organic frameworks (MOFs)

Metal–organic frameworks (MOFs) are a type of porous crystalline materials that have well-defined structures consisting of one-, two-, or three-dimensional networks (Omer and Ali 2022). Yaghi et al. were recognized as the trailblazers in the development of these compounds. MOFs are formed by combining inorganic metal ions/clusters with organic linkers by coordination bonding (Feng et al. 2020). Historically, MOFs have been produced by hydrothermal or solvothermal techniques that employ electrical heating on a small scale (Ameen et al. 2022; Hassan et al. 2024). Nevertheless, these techniques are laborious, requiring several hours to several days for the reaction to reach completion (Ameen and Omer 2024a). As a result, researchers have investigated several methods of synthesis to speed up the process and create crystals that are more consistent and smaller in size (Ameen and Omer 2024b; Bedair et al. 2024d). The available options include microwave-assisted (George et al. 2019), sonochemical (Zhang et al. 2019), and electrochemical approaches (Song et al. 2022). However, solvothermal/hydrothermal methods continue to be the most often used for MOF synthesis (Bedair et al. 2024b). Recent developments have brought forth ionothermal synthesis, continuous microfluidic synthesis, dry-gel conversion synthesis, and mechanochemical approaches to improve the environmental sustainability of MOF manufacture. The objective of these strategies is to develop MOF products that are more ecologically friendly. In addition, the synthesis of MOFs may be expanded to create hybrid structures by combining them with other organic–inorganic materials, such as core–shell structures and composites (Abdelhameed et al. 2023; Hammad et al. 2024; Mansour et al. 2024a). MOFs have notable benefits, such as large specific surface areas, extremely high porosity of up to 90% free volume, customizable pore diameters, the ability to be modified after synthesis, and outstanding heat stability (Naser et al. 2023). Their interior surface areas can surpass 6000 m^2^/g, indicating their promise for many applications. Due to their remarkable characteristics, as previously noted, MOFs have found applications in several fields including medicine, remote sensing, wastewater treatment, air purification, and renewable energy sources (Mansour et al. 2024d; Bedair et al. 2024a). Furthermore, MOFs are regarded as a significant category of materials for the purposes of energy storage, CO_2_ adsorption, alkane/alkene separation, and catalysis. Nanotechnology is a very active area of study in the realm of materials science. Moreover, MOFs are categorized as third-generation antibacterial substances with a specific structure (Gangalla et al. 2021). According to reports, MOFs have a regulated ion release efficiency in addition to the advantages described above, as compared to traditional bactericidal components. Consequently, MOFs have great potential in several areas such as food preservation and antibacterial coatings (Shen et al. 2020). Jo et al. (2019) synthesized four 3D Cu-MOFs and assessed their antibacterial efficacy against five bacterial strains: *Escherichia coli*, *Staphylococcus aureus*, *Klebsiella pneumoniae*, *Pseudomonas aeruginosa*, and Methicillin-resistant *Staphylococcus aureus*.

**Fig. 2:**
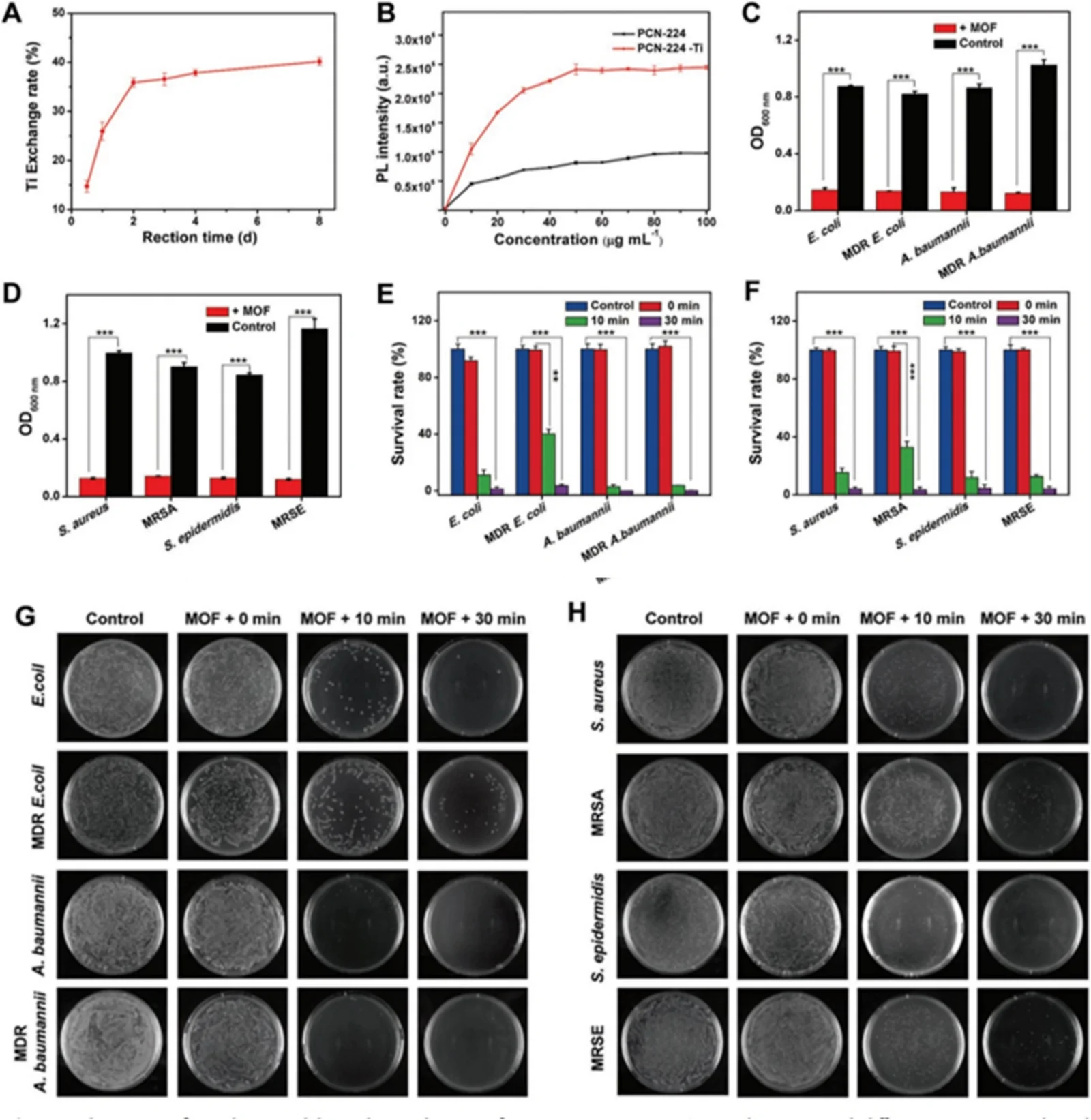
The process of titanium (Ti) exchange and the antibacterial activity of PCN-224(Zr/Ti) in a controlled laboratory environment. **A** Investigation of the rate of titanium exchange using the titanium inclusion method at different response times. **B** The DCFH PL spectra were obtained by exposing PCN-224 (black) and PCN-224(Zr/Ti) (red) to visible light for 3 min. These spectra were used to identify the formation of ROS from varied concentrations of PCN-224 and PCN-224(Zr/Ti). **C, D** Gram-negative and Gram-positive bacteria samples were subjected to visible light for a duration of 30 min. After 24 h, the treated and untreated bacteria were assessed using PCN-224(Zr/Ti). **E, F** The study investigated the survival rates of Gram-negative and Gram-positive bacteria when exposed to irradiation while being cultivated with PCN-224(Zr/Ti) at a concentration of 50 µg mL.^−1^. **G, H** Images of Gram-negative bacteria (*E. coli*, MDR *E. coli*, *A. baumannii*, and MDR *A. baumannii*) and Gram-positive bacteria (*S. aureus*, *MRSA*, *S. epidermidis*, and *MRSE*) were taken after being subjected to radiation for 0, 10, and 30 min. The bacteria were exposed to the radiation in an incubation chamber containing PCN-224(Zr/Ti). For comparison, the bacterial samples did not undergo treatment with PCN-224(Zr/Ti). There are two noteworthy *p*-values, one below 0.01 and the other over 0.001 (image taken with permission from Chen et al. (2020))

The antibacterial activity was assessed by obtaining the minimal bactericidal concentration (MBC) values against these five microorganisms. The results showed that all four produced Cu-MOFs exhibited identical antibacterial activity against all five tested strains of bacteria. The study conducted by Jo et al. (2019) revealed that the MBC for all Cu-MOFs was determined to be 20 µg/mL. This study found that the robust 3D frameworks with Cu-based surface active sites were more effective in inactivating different types of bacteria compared to the leached Cu^2+^ ions. This resulted in a reduction in the cytotoxicity produced by the leached metal ions. Hence, doing additional research on the correlation between the physical and chemical characteristics of contact-killing surfaces and their antibacterial efficacy would facilitate the creation of innovative materials with potential applications against various bacterial strains (Jo et al. 2019). Chen et al. (2020) conducted a study where they produced MOFs as a non-antibiotic agent for photodynamic therapy (PDT) of chronic wounds infected by multidrug-resistant (MDR) bacteria. The MOFs were used without any additional antibacterial chemicals, besides the documented characteristics of PDT, such as its low likelihood of drug resistance and minor safety concerns. Chen et al. (2020) conducted a study where they created NPs of MOFs (PCN-224) that were modified with titanium using a simple cation exchange method. The findings showed that the prepared bimetallic MOF, PCN-224(Zr/Ti), had excellent photocatalytic capabilities in producing reactive oxygen species when exposed to visible light. This attribute is responsible for its effective antibacterial properties. Figure 2 below depicts the mechanism of Ti exchange and the antibacterial activity of PCN-224(Zr/Ti) in vitro. Therefore, the research on MOFs for antibacterial applications remains vital and ongoing. Recent advancements in the development of MOFs have resulted in the creation of new types of MOFs that exhibit enhanced stability and do not release harmful cations. Additionally, these MOFs possess improved bactericidal characteristics. As a result, they have the potential to be excellent candidates for combating MDR bacteria (Chen et al. 2020).

Wang et al. (2020) conducted research where they created Cu-MOFs (HKUST-1) and integrated them into electrospun chitosan/polyvinyl alcohol (HKUST-1/chitosan/PVA) fibers. The fibers demonstrated exceptional antibacterial efficacy against *Escherichia coli* and *Staphylococcus aureus*, attaining an impressive antibacterial efficiency of 99%. The combined antibacterial action of HKUST-1 and chitosan played a role in this activity by establishing a germ-free environment for wounds and inhibiting infection. Therefore, the findings provided evidence that HKUST-1/chitosan/PVA fibers promoted faster wound healing with decreased inflammation in comparison to the other groups. The significance of antibacterial activity in wound dressings cannot be emphasized enough, considering that wounds often come with bacterial infections that hinder wound healing and may result in consequences including fever, bacteremia, and other severe infections. This was confirmed by prior research that examined antibacterial activity using the approach of counting bacterial colonies (Wang et al. 2020). Integrating MOFs into electrospun fibers shows great potential for the regeneration of skin tissue. Moreover, outside the findings stated above, other research has shown the use of MOFs as antibacterial agents, as summarized in Table S1.

**Fig. 3:**
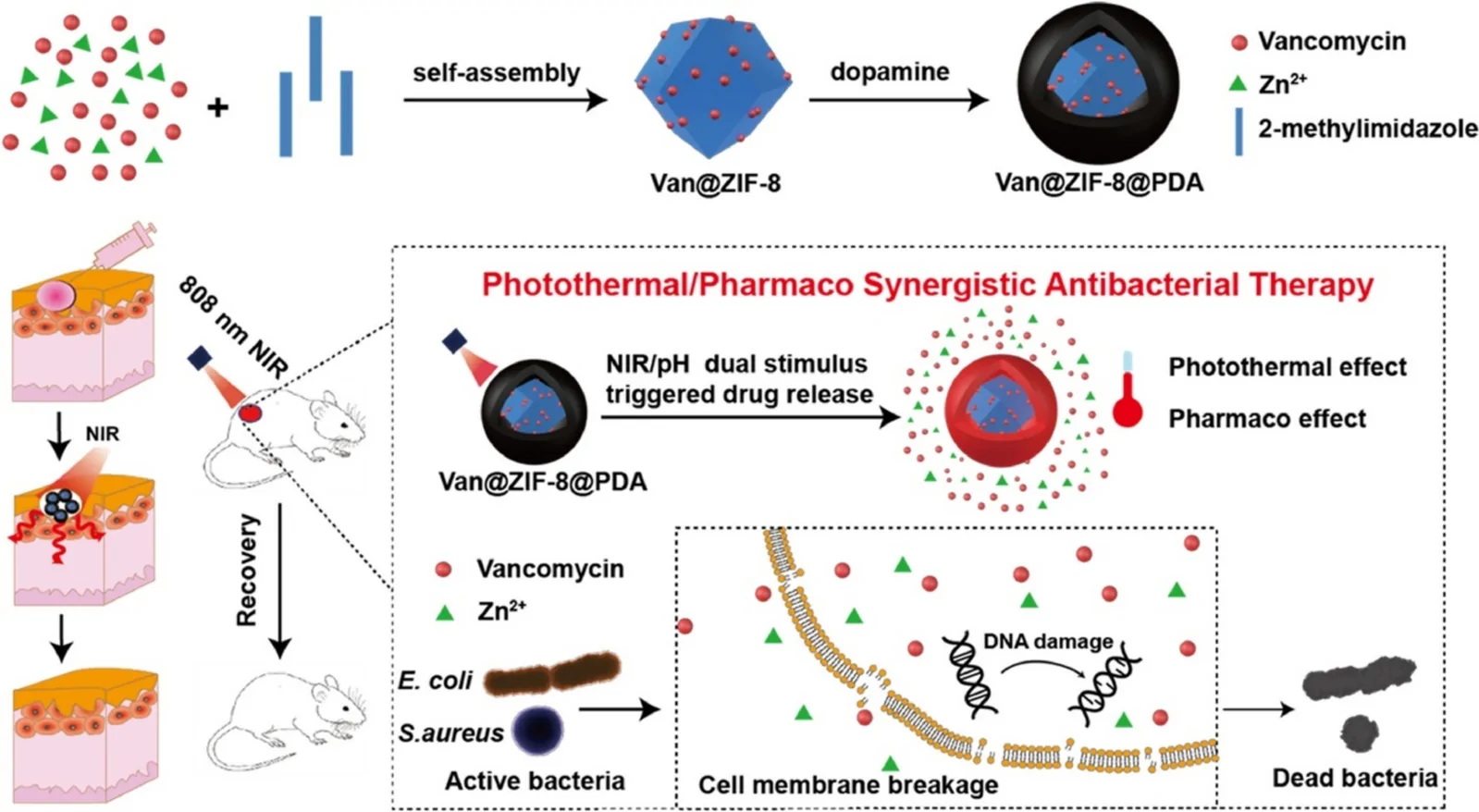
A schematic representation of a nanosystem based on MOFs that is responsive to two different stimuli (image taken with permission from Xiao et al. (2021))

Xiao et al. (2021) introduced an innovative approach to antibacterial therapy utilizing MOFs that respond to two distinct stimuli to amplify their antibacterial efficacy. The design of the nanosystem revolves around integrating photothermal and pharmacological antibacterial mechanisms within the MOF structure, offering a multifaceted approach to combat bacterial infections. The nanosystem is engineered with a MOF framework comprising a metal center (e.g., zinc or copper) and an organic linker, facilitating the encapsulation of antibacterial agents. It exhibits dual responsiveness to near-infrared (NIR) light and pH changes (Fig. 3). Upon exposure to NIR light, the MOF generated heat via a photothermal effect, which can induce damage to bacterial cells. Additionally, the nanosystem responds to pH changes, triggering the controlled release of the encapsulated antibacterial agents, particularly advantageous in acidic bacterial infection environments. The synergistic combination of photothermal and pharmacological mechanisms within the MOF-based nanosystem led to enhanced antibacterial activity. The photothermal effect disrupted bacterial cell membranes, rendering them more permeable to the released antibacterial agents, thereby augmenting their pharmacological efficacy (Xiao et al. 2021).

### Covalent organic frameworks (COFs)

Covalent organic frameworks (COFs) are a type of organic crystalline porous materials that have been extensively studied and are still in the early stages of development. They were first introduced by Yaghi et al. (1996) and are created through a process called reticular chemistry. COFs are constructed using building blocks that consist of light elements such as carbon (C), hydrogen (H), oxygen (O), nitrogen (N), or boron (B) atoms. The molecules are joined to one another by covalent bonds, which extend into either two or three dimensions (Abuzeid et al. 2021). In recent times, there has been notable attention towards the creation and use of 3D COFs, owing to their distinct porosity properties and higher performance in comparison to previously documented two-dimensional (2D) frameworks (Guan et al. 2020). Identifying optimal synthesis conditions for COFs is essential, especially for practical applications in the real world (Abuzeid et al. 2021). Solvothermal methods are often employed for the synthesis of COFs; however, other alternative strategies have been devised (Li et al. 2020), such as ionothermal, microwave-assisted, sonochemical, mechanochemical, and light-induced procedures.

Carbon-based organic frameworks (COFs) have advantageous characteristics that make them highly suitable for a wide range of applications. These qualities arise from the mix of light materials and organized frame structures, resulting in low densities, high surface areas, and amazing porosity. Additionally, COFs that are covalently connected have superior stability in comparison to well-recognized metal–organic frameworks (MOFs). Their capacity to mend themselves and the regulated bonding through thermodynamics lead to the creation of highly organized crystalline formations across extended distances. In addition, COFs exhibit exceptional chemical stability in organic solvents and resistance to harsh conditions such as acidic and basic environments. This is attributed to their purely covalent, metal-free nature, which allows them to maintain their ordered structures and crystallinity (Diercks and Yaghi 2017). Unlike MOFs, which depend on coordinative bonds, COFs do not rely on metal atoms. COFs possess a wide range of interesting characteristics, making them very suitable for many applications such as gas separation and storage, heterogeneous catalysis, chemical sensing, luminescence, electrical devices, drug administration, and energy storage and conversion (Abuzeid et al. 2021). In their study, Gendy et al. (2022) examined the suitability of COFs for biological activities due to their significant porosity and active spots. Although there have been some studies on the biological roles of COFs, this topic is still in its early stages of development. Furthermore, there is a scarcity of research on the antibacterial properties and drug resistance of COFs themselves. Recently, a number of studies have emphasized the antibacterial properties of COFs.

Salehi et al. (2023) investigated the antibacterial activity of COFs as a delivery system for the antibiotic trimethoprim against the bacteria *Escherichia coli* and *Staphylococcus aureus*. The study found that COFs loaded with trimethoprim exhibited enhanced antibacterial activity compared to trimethoprim alone (Fig. S1). The COF delivery system improved the solubility, stability, and controlled release of trimethoprim, leading to more effective inhibition of bacterial growth, suggesting that COFs have the potential as a drug delivery platform to enhance the therapeutic efficacy of antibiotics against problematic bacterial pathogens (Salehi et al. 2023). This effect is due to their rich pore structure, which enables COFs to load various antibacterial drugs; they can function as a drug delivery system with controlled release (Wang et al. 2024). Furthermore, COFs containing guanidinium or cationic groups, with cell membrane-binding abilities, also provide a promising feature for damaging the integrity and permeability of bacterial membrane (Wang et al. 2024). Moreover, COFs have customized structural blocks and chemical surfaces that not only enable the combination of multiple therapeutic approaches but also achieve selective material-target interactions, supporting the way for enhanced therapeutic efficacy for a wide range of conditions (Wang et al. 2024).

Gendy et al. (2022) did a study where they synthesized a new covalent organic framework (COF) called COFTDETA. This COF was created by condensing terephthaldehyde with diethylenetriamine using solvothermal processes (Gendy et al. 2022). The antibacterial activities of COFTDETA were assessed against Gram-positive bacteria (*Staphylococcus aureus* and *Enterococcus faecalis*) and Gram-negative bacteria (*Escherichia coli* and *Pseudomonas aeruginosa*), as shown in Table S2. Tobramycin, a wide-ranging antibiotic, was used as a control drug. The experiments indicated that COFTDETA showed substantial antibacterial efficacy against both bacterial strains, similar to that of tobramycin (Gendy et al. 2022). The effectiveness of COFTDETA in combating bacteria can be due to its electrostatic interactions and hydrogen bonding with the charged phospholipid bilayer of bacterial membranes. These interactions lead to significant antibacterial reactions, as seen in the study conducted by Gendy et al. (2022). Therefore, it is advisable to broaden the utilization of COFTDETA for antibacterial therapies in environmental settings. Dai et al. (2022) conducted a study where they produced COFs-AgNPs by immobilizing silver nanoparticles (AgNPs) on COFs by an in situ process. The COFs-AgNPs exhibited remarkable antibacterial efficacy against *Staphylococcus aureus* and *Escherichia coli*. The researchers created nanocomposite films by incorporating COFs-AgNPs into chitosan (CS) solution using the solution casting technique (Dai et al. 2022). The study found that the electrostatic interaction between COFs-AgNPs and CS increased the viscosity of the CS film-forming solution. The CS/COF-AgNP nanocomposite films showed significant antibacterial properties, indicating its possible use in food-active packaging (Dai et al. 2022).

Liang et al. (2022) effectively developed a composite material consisting of a covalent organic framework and carbon dots, referred to as COF@CD. This combination exhibited a high rate of charge transfer and indicated effective photocatalytic activity for antibacterial photocatalytic treatment. The researchers used *Escherichia coli* as a representative Gram-negative bacterium to assess the photocatalytic antibacterial effectiveness of the COF@CD nanocomposite, as described by Liang et al. (2022). The findings demonstrated that the combination of CD-integrated COF had a significantly improved antibacterial activity. Furthermore, it displayed a high level of safety for biological systems and had minimal harmful effects on human renal epithelial 293T cells (Liang et al. 2022). In their study, Hynek et al. (2018) described the synthesis of porphyrin-based covalent organic frameworks (COFs) via Schiff-based processes. The COFs exhibited substantial antibacterial activity against *Pseudomonas aeruginosa* and *Enterococcus faecalis* biofilms, which are two types of bacteria renowned for their propensity to build biofilms. These bacteria serve as models for Gram-negative and Gram-positive bacteria, respectively. The 3D-TPP coatings were the only ones that consistently retained their capacity to kill bacteria, demonstrating improved long-term effectiveness and stability. The antibacterial performance of the three distinct COF coatings is depicted in Fig. S2, indicating that porphyrinic COFs show great potential as candidates for developing antibacterial coatings for indoor use (Hynek et al. 2018).

Following a 48-h incubation period, a comparable experiment was conducted under 460 nm light (Fig. S2C). In order to assess the direct eradication of cells within the biofilms, the biofilms of *P. aeruginosa* and *E. faecalis* were formed by incubating them for 24 h in the absence of light and then subjecting them to 4 h of exposure to 460 nm light (Fig. S2D). The trials were conducted using the polymer covering in the absence of COFs. For all instances, the quantity of the biofilm was measured as a percentage of the surface area that was occupied by bacteria (*y*-axis). The experiments were analyzed using Student’s *t*-test, with findings that had a *p*-value of less than 0.01 considered statistically significant. In addition to the previously studied cases, several studies have reported on the antibacterial activity of COFs and COF hybrids. For more information on the antibacterial activity of COFs and COF hybrids, refer to Table S2.

### Quantum dots (QDs)

Alexei Ekimov made the initial discovery of QDs in the 1980s. QDs are semiconductor crystals at the nanoscale that have shown great potential in several scientific disciplines, with a particular focus on biology (Kargozar et al. 2020). These materials are spatially confined in all three dimensions at the nanoscale scale, enabling them to facilitate the movement of electrons (Singh et al. 2021). Conventional QDs, referred to as core nanocrystals, generally include two unique components: a core made of heavy metal and a shell made of a wide bandgap semiconductor (Wang et al. 2013). Nevertheless, these QDs are restricted by their unfavorable toxicity towards live cells, tissues, and organisms, mainly caused by the release of cadmium ions from the core (Liu et al. 2014). In order to overcome these constraints, researchers have produced a novel type of quantum dots called cadmium-free quantum dots (CFQDs) (Kargozar et al. 2020). Some examples of colloidal quantum dots (CFQDs) are silicon QDs (Si QDs), near-infrared QDs such as Ag_2_Se QDs and Ag_2_S QDs, carbon dots (C-dots), graphene QDs (GQDs), and Au/Ag/Cu clusters (Xu et al. 2016). Out of all these new QD systems, the carbon dots (C-dots) family has gained a lot of attention because of their outstanding electrical characteristics. C-dots have the ability to function as both electron donors and acceptors, leading to chemiluminescence and electrochemical luminescence. This makes them well-suited for use in optoelectronics, catalysis, and sensors.

QDs can be synthesized using physical, chemical, or biological means. Physical methods include manipulating matter without any chemical transformations. Chemical methods involve chemical processes that result in the synthesis of new compounds. Biological methods utilize live organisms to create QDs. Organometallic synthesis, which is one of the initial techniques employed for producing QDs, utilizes a “bottom-up” strategy to fabricate QDs with exceptional optical characteristics. Another often employed technique is aqueous solution-based synthesis, which is considered to be more environmentally benign in comparison to the organometallic approach. The biomolecule-templated approach is also regarded as a feasible technique for quantum dot (QD) synthesis (McMillan et al. 2002). Utilizing biomolecules such as nucleic acids, peptides, and polysaccharides as templates, this approach has emerged as a novel paradigm for constructing inorganic nanomaterials. Instances of this technique encompass QDs constructed by DNA templates (Wei et al. 2014b), protein cages generated from heat shock proteins, and genetically modified viruses (Kargozar et al. 2020). In addition, the synthesis of QD in live creatures is an innovative method. Cadmium sulfide quantum dots (CdS QDs) have been synthesized in many types of bacteria, including *Escherichia coli* (Depeursinge et al. 2010), *Bacillus megaterium*, and *Gluconacetobacter xylinus* (Kargozar et al. 2020).

**Fig. 4:**
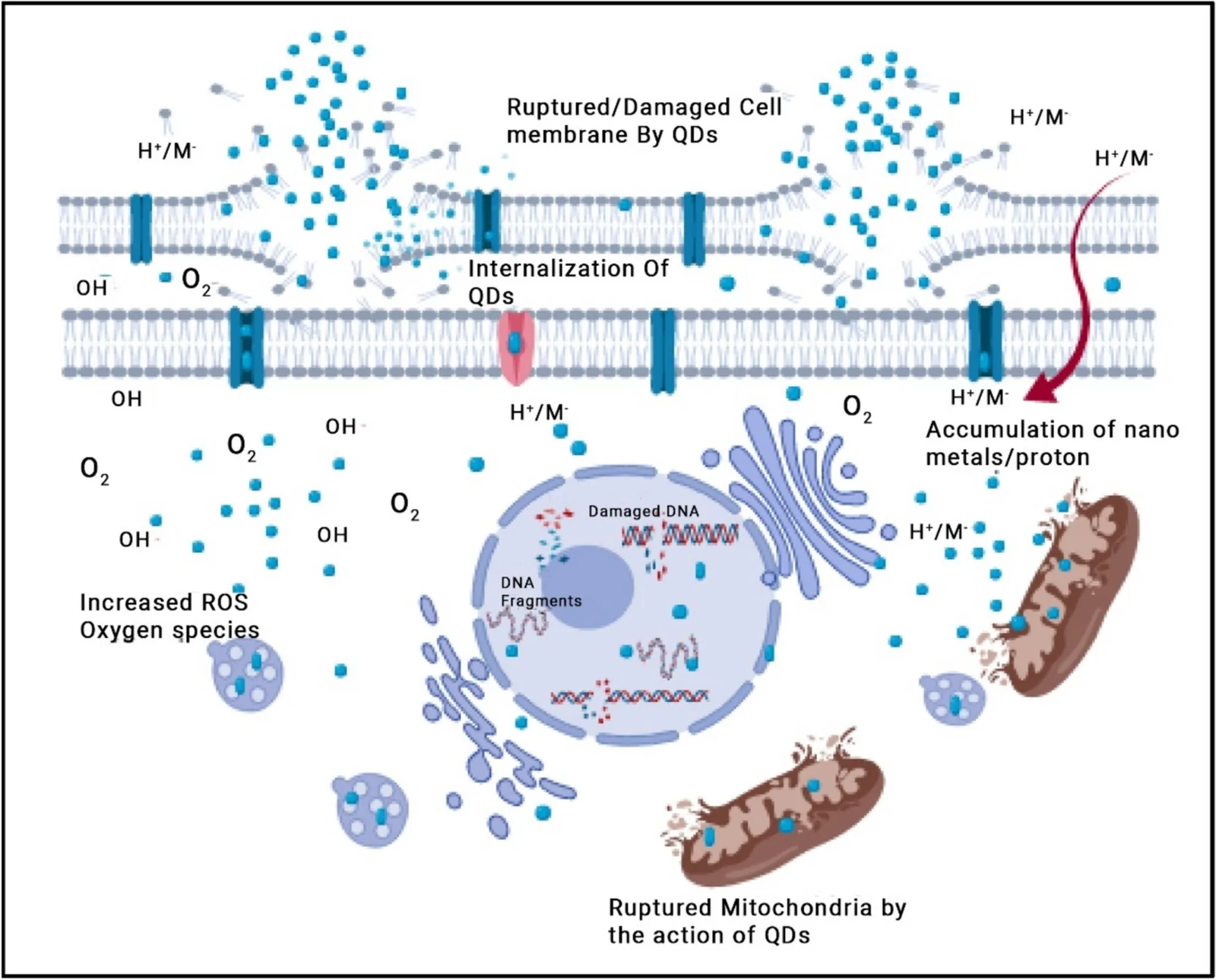
Quantum dots’ general antibacterial mechanism (image taken with permission from Rajendiran et al. (2019))

QDs provide several benefits, such as their compacted sizes ranging from 4 to 12 nm, the ability to adjust the size-dependent photoluminescent (PL) emission, a high extinction coefficient, and a high fluorescence quantum yield. In addition, QDs have exceptional resistance to photobleaching and fluorescence intermittency (Pal et al. 2015). QDs have received considerable interest in the fields of biology and medicine due to their use as luminous probes and labels in drug delivery and targeting, as well as their usage in DNA and oligonucleotide sensing (Kargozar et al. 2020). They are also utilized in many biological imaging techniques, such as molecular histopathology, flow cytometry-based detections, disease diagnostics, and general biological imaging. Furthermore, QDs are essential in the context of antibacterial applications when exposed to light because they generate reactive oxygen species (ROS) (Tian et al. 2019). Nevertheless, the practical use of these materials is impeded by many intrinsic flaws. These include their tendency to aggregate together, surface imperfections that make them less stable than their larger counterparts, and their strong photoluminescence, which causes the fast recombination of photoexcited charge carriers (Yamashita et al. 2015). An effective technique to tackle these problems is to load QDs onto 2D materials or encapsulate them into metal–organic frameworks (MOFs) to create hybrid nanostructures. These properties are improved: stability, dispersibility, and photocatalytic activity (Yamashita et al. 2015; Wang et al. 2021). In the work conducted by Wang et al. (2021), they synthesized a nanocomposite called QDs@ZIF-8, which showed a remarkable ability to kill bacteria such as *Escherichia coli* and *Staphylococcus aureus* when exposed to visible light. This finding was also supported by Kargozar et al. (2020). The nanocomposite’s antibacterial properties are due to the effective transfer of electrons at the interface between ZIF-8 and ZAIS QDs, resulting in the production of a higher amount of ROS. The antibacterial method entails the eradication of the bacterial cell membrane, decomposition of internal macromolecules including DNA and proteins, and oxidation of glutathione (Wang et al. 2021). The study presents an innovative and encouraging approach to develop photocatalytic disinfection materials using MOFs and semiconductor quantum dots (Wang et al. 2021). Figure 4 provides a concise overview of the overall antibacterial mechanism of QDs.

In a study by Liu et al. (2018), ZnO/graphene quantum dot (GQD) nanocomposites were produced using a facile hydrothermal method. The antibacterial activity of the ZnO/GQD nanocomposites was evaluated against *Escherichia coli* by determining the MIC and the reduction in the number of bacterial colonies using a standard plate count method. The activity was found to be significantly enhanced under UV photoirradiation compared to ambient light. This enhancement is likely due to the increased generation of ROS under UV light, with minor contributions from membrane damage, as evidenced by electron paramagnetic resonance and fluorescence microscopic measurements (Liu et al. 2018). This study underscores the importance of functional nanocomposites based on semiconductor nanoparticles and graphene derivatives in developing effective bactericidal agents (Liu et al. 2018). In a more recent study by Khan et al. (2024), type-II QDs were developed, and their antibacterial activity was evaluated against two Gram-negative bacteria: *Pseudomonas aeruginosa* and *Escherichia coli*. The results indicated that InP/ZnO QDs simultaneously generate ROS, including hydroxyl (^•^OH) and superoxide (O_2_^•^ −) radicals, while type-I sulfide-capped InP/ZnS QDs only release O_2_^•^ − radicals. This novel nanostructure achieved effective inhibition of bacterial growth, reaching 99.99 and 70.31% under low-intensity green light illumination of 5 mW/cm^2^. These findings highlight the potential of type-II QDs as a new nanostructure for developing effective antibacterial agents against drug-resistant pathogens (Khan et al. 2024). Several other studies have also reported on the antibacterial activity of QDs and QD hybrids. For more detailed information, refer to Table S3.

### Carbon quantum dots (CQDs)

Carbonaceous and carbon-based nanomaterials have lately attracted considerable interest due to their intriguing characteristics (El-Shabasy et al. 2021; Kannouma et al. 2024a). CQDs are a recently discovered type of fluorescent nanostructures known as quantum dots, which are utilized in a wide range of applications. CQDs are carbon nanoparticles that have a zero-dimensional (0D) structure and are generally less than 10 nm in size (Molaei 2020). A variety of fluorescent carbon dots, including graphene quantum dots (GQDs), polymer dots (PDs), carbon nanotube (CNT) dots, nanodiamonds (NDs), and CQDs have been synthesized thus far (Molaei 2020). The manufacture of CQDs may be achieved using two primary methods: top-down and bottom-up techniques (El-Shabasy et al. 2021). CQDs are generated using chemical and physical cutting processes, such as electrochemical processes (Zhang et al. 2013), laser ablation (Cui et al. 2020), and high-energy ball milling, in top-down approaches. Bottom-up approaches are seen as more convenient and well-suited for the efficient and extensive manufacturing of CQDs (El-Shabasy et al. 2021). These approaches involve the synthesis of carbon dots from suitable chemical precursors using specified conditions such as combustion, hydrothermal, thermal, and ultrasonic irradiation (Tang et al. 2012; Zhang et al. 2017). These technologies need a reduced number of carbon sources and are typically more economical.

The bottom-up approach is frequently favored over top-down methodologies due to many drawbacks of the latter, such as exorbitant expenses, prolonged processing durations, and rigorous criteria (Sharma and Das 2019). Nevertheless, the majority of synthesis processes are characterized by their multistep nature, necessitating the use of harsh temperatures and expensive carbon sources that may potentially have harmful effects. Consequently, there is a growing preference for green synthesis (El-Shabasy et al. 2021). Green chemistry, a prominent field within the realm of chemistry, provides notable benefits such as enhanced safety, environmental friendliness, and the eradication of harmful substances (Abdallah et al. 2021; Mabrouk et al. 2023). Carbon dots may be produced from several natural sources, such as chicken eggs, animals, and numerous plant species including fruits and vegetables, using environmentally friendly chemical techniques that can take place under normal settings (Mehta et al. 2015; Sharma et al. 2018; Kannouma et al. 2024b). Waste resources such as scrap paper and frying oil have also been utilized (Wei et al. 2014a). The manufacturing procedures can be accomplished using various techniques, such as hydrothermal/solvothermal synthesis, microwave-assisted polymerization, pyrolysis, and carbonization. These methods are often employed in the synthesis of carbon dots, offering several benefits (El-Malla et al. 2022; Ahmed Abdel Hamid et al. 2023; Elshenawy et al. 2023).

**Fig. 5:**
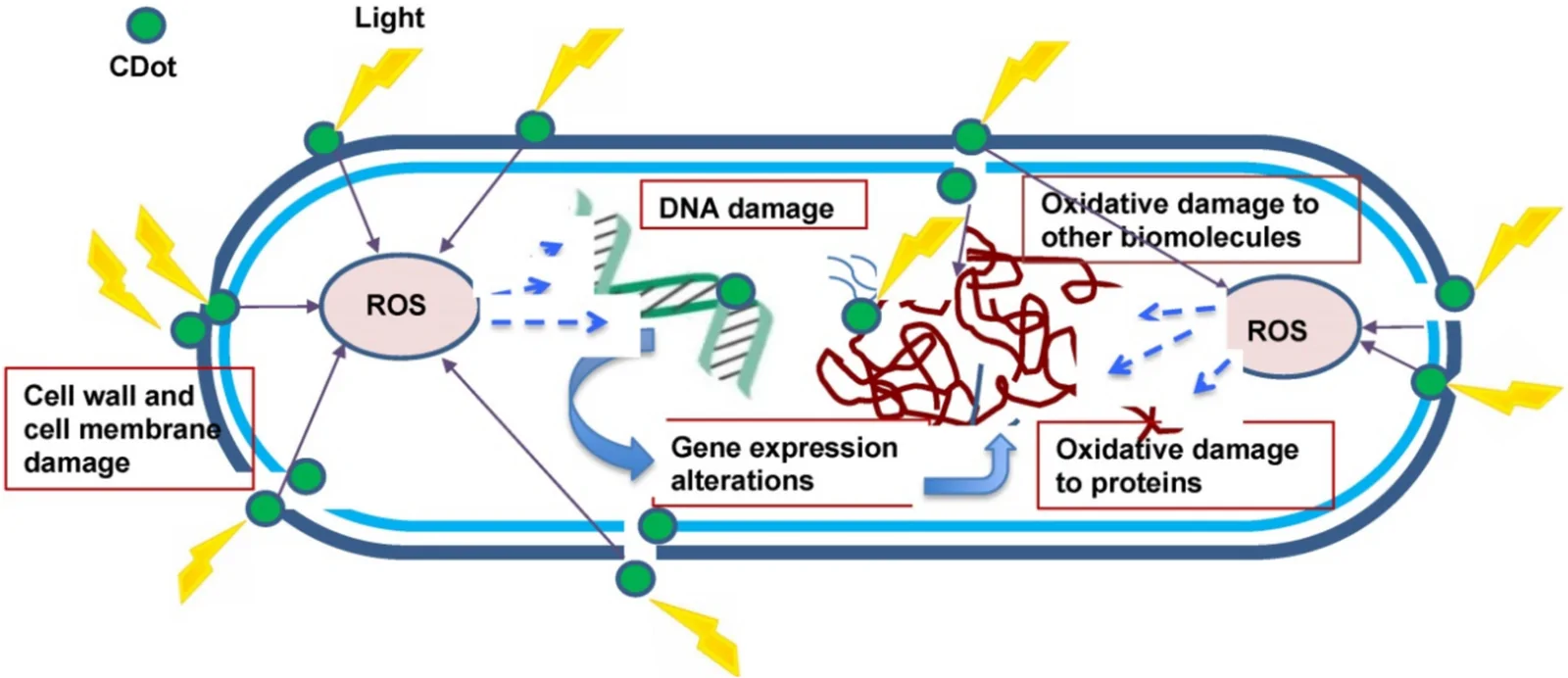
Diagram illustrating the mechanism via which photoactivated antibacterial properties of CQDs work (image taken with permission from Dong et al. (2020))

CQDs possess several advantageous qualities that contribute to their extensive utilization throughout various domains (Elshenawy et al. 2022; Mansour et al. 2024c; Alomar et al. 2024). Notable characteristics of CQDs include favorable photoluminescence (PL) properties, as the PL can be adjusted and extends from deep ultraviolet to near-infrared (NIR) wavelengths (Molaei 2020). Additionally, CQDs can be synthesized easily using cost-effective methods and inexpensive starting materials and exhibit low toxicity levels and chemical stability (El-Malla et al. 2021; Elshenawy et al. 2022; Hamid et al. 2024). Moreover, the inclusion of functional groups such as hydroxyl, epoxy, carbonyl, and carboxyl on the carbon core of CQDs grants them the ability to dissolve in water and provides a convenient framework for straightforward modification with various substances (Yuan et al. 2016; Abdella et al. 2024; Kamal et al. 2024). Moreover, the photoluminescent properties of CQDs allow them to produce light in the near-infrared (NIR) part of the electromagnetic spectrum. This makes them suitable for many applications such as bioimaging, cancer treatment, drug administration, and photoacoustic (PA) imaging. In addition, CQDs possess some benefits that are not commonly found in standard QDs and organic dyes. These advantages include photo-stability and resistance to blinking and photobleaching (Yuan et al. 2016). Figure 5 illustrates the mechanisms by which CQDs act as antibacterial agents.

Lately, there has been an increased focus on the use of nanomaterials in antibacterial and associated applications (Zhao et al. 2022). A recent study conducted by Kang et al. (2024) explored the application of CQDs as antibacterial agents synthesizing innovative CQDs by utilizing discarded coffee grounds (DCG) as a source of biomass. They employed microwave treatment in the process. The CQDs that were created showed antibacterial activity that was driven by visible light and increased as the pH fell. The antibacterial properties of CQDs were assessed using *Staphylococcus aureus* and *Escherichia coli* O157:H7 as representative models for Gram-positive and Gram-negative bacteria, respectively. Figure S3 demonstrates a progressive drop in the negative zeta potential (mV) values of CQDs and pathogenic bacteria as the pH decreases. As a result, pathogens had an enhanced absorption of CQD since the repulsive force between them decreased. In addition, the activity of superoxide dismutase and catalase enzymes, which are responsible for removing ROS in cells, dropped as the pH declined. In addition, the presence of CQDs resulted in a rise in the production of ROS within cells when exposed to visible light, with the quantity of ROS created being higher as the pH declined (Kang et al. 2024). These findings indicate that CQDs produced from DCG were used in conjunction with malic acid to effectively clean fresh vegetables. The visible light treatment of a mixture of CQDs and malic acid, both synthesized from DCG, was used to observe the washing effect on artificially contaminated apple samples. The contamination was done using *Escherichia coli* O157:H7, a significant pathogenic bacterium found in fresh produce. This process is illustrated in Fig. S4. The study validated that the amalgamation exhibited a remarkable control efficacy against harmful bacteria, indicating that this approach might be efficiently employed in the food business (Kang et al. 2024).

Hao et al. (2021) conducted a study where they created positively charged CQDs (p-CQDs) and utilized them as antibacterial agents with a wide range of effectiveness. The results indicated that p-CQDs exhibited significant antibacterial efficacy against all the pathogenic microorganisms tested. The investigation of the antibacterial mechanism of p-CQDs revealed that p-CQDs with small sizes and functionalized with -NH_2_ and -NH groups exhibited a robust adhesion behavior on the bacterial cell membrane. In addition, the introduction of p-CQDs resulted in conformational alterations in the bacterial genes and the production of ROS. An assessment of safety revealed that p-CQDs did not induce observable drug resistance or hemolysis (Hao et al. 2021). In addition, p-CQDs efficiently enhanced the antibacterial therapy of wounds contaminated with a combination of *Staphylococcus aureus* and *Escherichia coli* in rats, demonstrating little toxicity in vivo. These findings indicate that p-CQDs have the potential to be used as an antibacterial treatment for actual wound healing scenarios, particularly for complicated and drug-resistant bacterial infections (Hao et al. 2021).

Geng et al. (2021) conducted research that revealed that the antibacterial and osteogenic properties of CQDs may be controlled by modifying their surface charge. p-CQDs had significant antibacterial effects against MDR bacteria and inhibited the production of biofilms. Conversely, CQDs with a negative charge (n-CQDs) greatly enhanced the process of bone repair. In order to avoid the clustering of p-CQDs and n-CQDs in scaffolds, the authors created essentially neutral p-CQD/WS2 hybrids by applying a layer of p-CQDs over WS2 nanosheets. Afterwards, GelMA hydrogel scaffolds were created by co-encapsulating multifunctional biodegradable p-CQD/WS2 and n-CQDs. Implanting multifunctional p-CQD/WS2/n-CQD/GelMA hydrogel scaffolds in a craniotomy defect model infected with methicillin-resistant *Staphylococcus aureus* (*MRSA*) led to nearly complete repair of the infected bone defect. This resulted in a new bone area of 97.0 ± 1.6% after 60 days (Geng et al. 2021). The work suggests that a surface charge management approach may be used to develop biomaterials that possess both antibacterial and osteogenic properties, which can be beneficial for treating bone defects that are infected (Geng et al. 2021). Furthermore, several other research papers have documented the utilization of CQDs and CQD hybrids as agents with potential antibacterial properties. Table S4 provides comprehensive data on the use of CQDs and CQD hybrids as antibacterial agents against a range of pathogens.

### Ionic liquids (ILs)

Paul Walden defined ILs as organic salts that have melting points below 100 °C. These chemicals generally exist in a liquid state when exposed to ambient temperature. Typically, they are composed of bulky, asymmetrical organic cations, and complex organic or inorganic anions (Ventura et al. 2017; Danielson et al. 2018; Abdelaziz et al. 2021). ILs, or ionic liquids, are regarded as very favorable and environmentally friendly solvents, offering a substitute for routinely employed volatile organic solvents (Singh 2019; Abdallah et al. 2022; Abdelaziz et al. 2023). A range of methods have been devised to create a sequence of ILs, such as microwave (MW) radiation, sonication, macrocyclic ILs, ring opening, acid–base neutralization, crown ethers, power ultrasound (US), and others (Lévêque et al. 2002; Ratti 2014; Singh and Savoy 2020). Out of these approaches, microwave irradiation and ultrasound-assisted treatments provide several benefits. The microwave irradiation method is rapid and utilizes a secure heat source in the absence of solvents, resulting in high atom efficiency by enhancing product selectivity and achieving optimal outcomes within a brief reaction time. The ultrasonic approach is a sustainable technology employed for the synthesis of ILs without the use of solvents, resulting in significant product yields (Singh and Savoy 2020). The approach described is very efficient in the interfacial layers of two liquids that do not mix, resulting in increased reaction rates and improved material transformation, all while minimizing the time required for the reaction (Singh and Savoy 2020). In order to enhance energy efficiency and promote environmentally friendly practices, it is advisable to utilize techniques such as microwave irradiation and ultrasound-assisted processes for the synthesis of ILs (Singh and Savoy 2020).

ILs have extensive uses in several fields such as electrochemistry, separation and extraction processes, as solvents and catalysts, and in analytical, physical, synthetic, biological, and engineering chemistry, among others (Roy et al. 2015; Bedair et al. 2024c). In addition, the recently uncovered biological functions of ionic liquids have attracted attention from biochemists, microbiologists, and medical scientists. This is primarily due to their antibacterial properties, which offer new possibilities for tackling the difficulties posed by antibiotic-resistant pathogens (Nikfarjam et al. 2021).

**Fig. 6:**
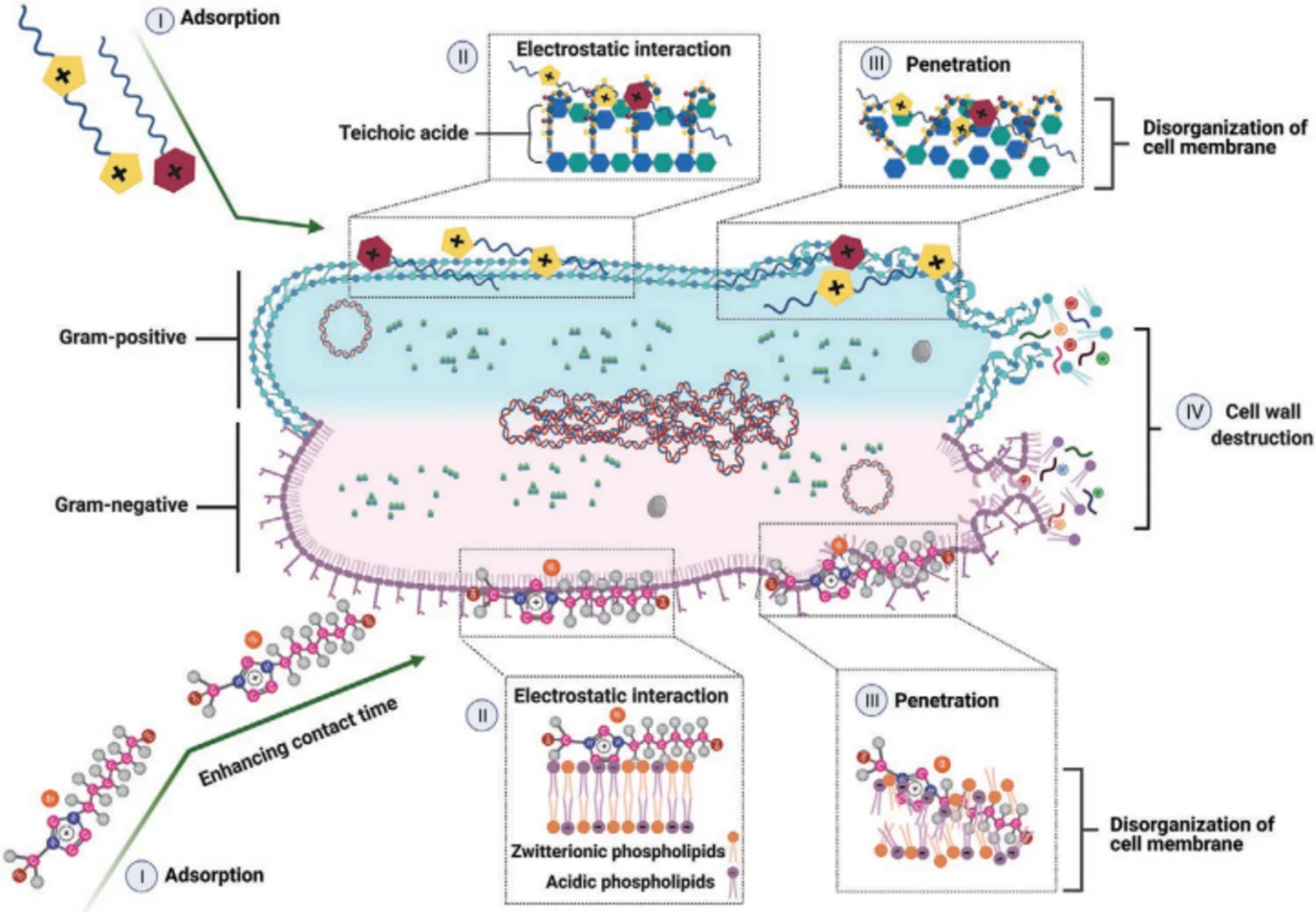
The process by which ionic liquids cause the disintegration of cell walls in both Gram-negative and Gram-positive bacteria is referred to as cell wall degradation. Bacterial cell death can be categorized into four stages: (I) adsorption, which refers to the process of ionic liquids approaching the bacterial cell wall/membrane; (II) electrostatic interaction, which involves the interaction between the functional groups of the zwitterionic phospholipid bilayer and the ionic liquids; (III) penetration, which describes the entry of the ionic liquid into the bacterial cell, leading to the disruption of the cell membrane; and (IV) cell wall destruction, which results in cell lysis (image taken with the permission of Nikfarjam et al. (2021))

ILs exert their mode of action by traversing bacterial membranes, penetrating the cytosol, and modifying the membrane properties of the bacterial cell wall. The primary causes contributing to the antibacterial characteristics of ILs are linked to various aspects. Firstly, ILs undergo adsorption due to their attraction to cell membranes. Additionally, they participate in electrostatic interactions by inactivating bacterial membrane proteins and interacting with membrane phospholipids. Furthermore, ILs have the ability to infiltrate the cell membrane, causing the breakdown and disruption of the phospholipid bilayer. This results in the release of intracellular cytoplasm. Ultimately, the process concludes with the disintegration of the cell wall, leading to cell lysis. Figure 6 depicts the process of cell wall degradation in both Gram-negative and Gram-positive bacteria caused by ionic liquids (Nikfarjam et al. 2021).

Multiple researches have documented the utilization of ILs in diverse applications. A research conducted by Takahashi et al. (2019) investigated a medication delivery method that included the IL 1-butyl-3-methylimidazolium hexafluorophosphate [BMIM][PF_6_] into chitosan-modified polymeric nanoparticles. An assessment was conducted to determine the antibacterial properties and cytotoxic effects of three different types of polymeric nanoparticles that included ILs. The findings demonstrated that Sol nanoparticles, which were modified with chitosan and integrated with IL, displayed the most potent antibacterial effects against biofilm bacterial cells. These nanoparticles also exhibited little toxicity (Fig. S5). This study revealed that the utilization of ILs in the preparation of polymeric nanoparticles significantly improved their ability to kill bacteria and their ability to remain in the body, even without the presence of a medicine. The results indicated that these nanoparticles have the potential to be utilized in the prevention and early treatment of periodontal disease (Takahashi et al. 2019). Another study also investigated the impact of these ILs on the antibacterial characteristics of LVF by creating different combinations of ILs with LVF. Moreover, Siddiquee et al. (2021) discovered a noteworthy increase in the antibacterial effectiveness of LVF when it was coupled with ILs. This enhancement was particularly noticeable when ILs with longer alkyl chain lengths were used (Siddiquee et al. 2021). In addition, the researchers assessed the antibacterial properties of these imidazolium-based ILs against *Escherichia coli* and *Staphylococcus aureus*, which are examples of Gram-negative and Gram-positive bacteria, respectively (Siddiquee et al. 2021). Besides the previous studies, there are several studies that reported the antibacterial activities of ILs. Table S5 illustrates more detailed information on ILs as antibacterial agents.

However, there are some limitations to using ILs, as it is challenging to ensure that all ILs or their metabolic products and byproducts formed during decomposition or degradation retain favorable physicochemical properties, such as negligible vapor pressure. This raises concerns about their potential to enter the environment and affect living and nonliving entities, particularly with long-term exposure in humans (Mondal and Bera 2022). Additionally, the toxicities including ecotoxicity, cytotoxicity, and health-hazard studies of the ILs have indicated that the popularly known concept of the low toxicity of ILs is inaccurate, mistaken, and misleading. However, the level of toxicity of the ILs is not potentially severe and life-threatening (Mondal and Bera 2022).

### Deep eutectic solvents (DES)

Deep eutectic solvents (DESs) are a novel category of solvents that possess a liquid state at or around room temperature (Abbott et al. 2003). These solvents, initially documented in the early twenty-first century, comprise a combination of two or more solid constituents, leading to a reduced melting point in comparison to the original substances. DESs are regarded as a favorable substitute for conventional organic solvents and ILs owing to their numerous benefits, including reduced volatility, diminished flammability, simplified manufacture, non-toxicity, environmentally friendly characteristics, biodegradability, and overall harmless nature. Furthermore, the substances employed in their formulation are inexpensive (Marchel et al. 2022).

DESs are generally utilized as environmentally friendly solvents in diverse chemical sectors, owing to their advantageous characteristics. Recently, they have also been utilized as very favorable liquids in the cosmetic, food, pharmaceutical, biotechnological, and biomedical sectors (Marchel et al. 2022). Nevertheless, there are several worries regarding their toxicity and a scarcity of research on this subject. Several reports on the biocompatibility of certain solvents are conflicting. Hence, in order to enhance the utilization of DESs in the field of green and sustainable chemistry, more investigations on their effects on organisms and the implementation of suitable methodologies are imperative.

Aside from conventional DESs, a novel and promising category of environmentally friendly solvents called natural deep eutectic solvents (NADES) has been documented. These solvents have garnered considerable interest as substitutes for regular organic solvents due to their possible antibacterial properties (Al-Akayleh et al. 2022; Bedair et al. 2024e). NADES are recognized for their reduced toxicity and increased capacity to break down naturally. They are mostly made up of cholinium chloride, natural carboxylic acids, sugars, amino acids, and occasionally water (Choi et al. 2011). Typically, these environmentally friendly solvents are transparent liquids that lack color when kept at normal room temperature. They are commonly produced using methods similar to those used for conventional deep eutectic solvents (Al-Akayleh et al. 2022).

Both DES and NADES may be produced using six physical methods: heating and stirring, freeze-drying, evaporation, grinding, ultrasound-assisted synthesis, and microwave-assisted synthesis. The heating and stirring approach is widely employed because of its simplicity and cost-effectiveness (Florindo et al. 2014; Castro et al. 2018). Vacuum evaporation is a method in which a combination of solvent components is heated at lower pressure to eliminate surplus water and achieve a uniform liquid state (Dai et al. 2013). Ultrasound-assisted synthesis utilizes sound waves to induce cavitation, which facilitates the formation of these solvents (Rutkowska et al. 2017). Microwave-based techniques involve exposing precursors to microwave radiation, which induces molecular agitation and accelerates solvent production (Gomez et al. 2018). Different researchers have utilized these methodologies, each implementing particular changes in processes and situations.

NADES, like DESs, possess several advantageous characteristics such as non-flammability, appropriate viscosity, biodegradability, straightforward environmentally friendly production, a broad range of polarity, low volatility, good thermochemical stability, compatibility with water, and minimal toxicity profiles. Moreover, they exhibit promising appropriateness as carriers for several drug delivery systems (Al-Akayleh et al. 2022). NADES have found extensive applications in several fields such as chemical and enzymatic processes (Dai et al. 2013; Nian and Li 2022), essential oil extraction (Rodríguez-Juan et al. 2021), nanocarriers for drug delivery (Zainal-Abidin et al. 2019), and as antioxidant and antibacterial agents (Radošević et al. 2018).

Both DESs and NADES possess antibacterial properties against a range of microbes. Oliveira et al. (2023) conducted a study on the antibacterial action of NADES based on menthol (Me) and saturated free fatty acids (FFA). The antibacterial efficacy was assessed against methicillin-resistant S*taphylococcus aureus* (*MRSA*), *Candida albicans*, and *Escherichia coli*, which respectively represent Gram-positive bacteria, fungi, and Gram-negative bacteria. The antibacterial assays consisted of three methods: the disk diffusion method was used for the initial screening of NADES antibacterial activity, the microbroth dilution technique was employed to determine the minimum inhibitory concentrations of these NADES against various pathogens, and the Alamar Blue assay was utilized to evaluate the removal/detachment of biofilms. The findings demonstrated that the use of Me and FFA in DESs successfully inhibited the attachment and growth of microorganisms (Oliveira et al. 2023). Furthermore, these DESs have favorable physicochemical characteristics. The thermoresponsiveness of a combination of menthol and lauric acid, with a molar ratio of 4:1, exhibits significant promise for surface modification in wound dressing applications, particularly at temperatures close to the body’s normal temperature. Hence, the utilization of DES formulated with Me and FFA presents a viable option as antibacterial and anti-inflammatory agents, with the prospect of being implemented in clinical settings (Oliveira et al. 2023).

Akbar et al. (2023) conducted a study where they synthesized several DESs and assessed their antibacterial effects on Gram-positive bacteria models such as *Streptococcus pyogenes*, *Bacillus cereus*, *Streptococcus pneumoniae*, and methicillin-resistant *Staphylococcus aureus*. Moreover, *Escherichia coli* K1, *Klebsiella pneumoniae*, *Pseudomonas aeruginosa*, and *Serratia marcescens* were employed as representatives of Gram-negative bacteria (Akbar et al. 2023). Their evaluation was conducted using conventional plate counting assays (Akbar et al. 2023). The DESs that were generated were further assessed for their cytopathic effects on human cells utilizing lactate dehydrogenase tests. The antibacterial tests demonstrated that DESs synthesized from a mixture of methyl-trioctylammonium chloride and glycerol, as well as DESs synthesized from methyl-trioctylammonium chloride and fructose at a 2 µL dosage, displayed a wide range of antibacterial effects, with the greatest bactericidal activity. The study presented evidence that DESs based on methyl-trioctylammonium chloride, when combined with various hydrogen-bond donor molecules, had favorable features for inhibiting bacteria in a range of infections. Furthermore, these DESs had no harmful effects on human cells, indicating their potential as safe and effective treatments (Akbar et al. 2023). The findings provide evidence that methyl-trioctylammonium chloride-based deep eutectic solvents (DESs) have the potential to be used as chemotherapeutic agents against bacteria (Akbar et al. 2023).

Furthermore, in the study by Al-Akayleh et al. (2022), the potential antibacterial effect of menthol, capric acid, and Solutol™ and their associated eutectic systems were evaluated. *Staphylococcus aureus* ATCC 6538 and *Bacillus subtilis* ATCC 6633 were used as models of Gram-positive bacteria, while *Escherichia coli* ATCC 8739 and *Pseudomonas aeruginosa* ATCC 9027 served as models of Gram-negative bacteria, and the yeast *Candida albicans* ATCC 10231 represented fungi (Al-Akayleh et al. 2022). The results indicated that the synthesized NADES and self-emulsifying drug delivery systems (SEDDS) exhibited promising antibacterial activity against *Staphylococcus aureus* and *Candida albicans*, with good activity against *Pseudomonas aeruginosa* (Al-Akayleh et al. 2022). Notably, these microorganisms are major pathogens associated with skin, ear, and eye infections, often carrying resistance factors to many available antibiotics. However, they rarely develop resistance against NADES based on saturated fatty acids due to the nonspecific mechanism of action (Silva et al. 2019). These findings underscore the potential use of NADES as preservatives in pharmaceutical preparations, in treating infections caused by these microorganisms, and in reducing the emergence of antibiotic-resistant strains. In addition to the aforementioned studies, several others have reported on DESs and NADESs as antibacterial agents. More detailed information on these studies is provided in Table S6.

### Layered double hydroxides (LDHs)

Layered double hydroxides (LDHs), often referred to as anionic clays or hydrotalcite-like materials, are a class of inorganic nanostructured materials having a 2D structure and basic properties (Janani et al. 2021). The materials comprise brucite-like layers with a positive charge, anions that compensate for the charge, and solvation molecules in the interlayer area (Abdallah et al. 2023). The composition of LDHs closely resembles that of mineral hydrotalcite ([Mg_6_A_l2_(OH)_16_]CO_3_·4H_2_O), which was first identified in 1842 (Jijoe et al. 2021). The synthesis of LDHs was initially conducted by Feithnecht in 1942, who referred to the compound as a “double-sheeted structure” (Mohapatra and Parida 2016). The formula for LDHs is M^2+^_1-x_M^3+^_x_(OH)_2_.A^n−^_x/n_.zH_2_O, where M^2+^ represents a divalent metal cation, M3 + represents a trivalent metal cation, and An − represents an anion (Mishra et al. 2018). The cations are accountable for the layers that carry positive charges. The density of these charges is directly related to the ratio *x* of trivalent metals, where *x* is equal to M^2+^/(M^2+^ + M^3+^), and usually falls between 0.2 and 0.33. The interlayers of LDHs consist of anionic species and water molecules, which play a crucial role in maintaining the stability of LDHs (Jijoe et al. 2021).

LDHs may be produced by combining different combinations of metal (II) and metal (III) cations, with the anions between the layers being carbonate, sulfate, or chloride. Nevertheless, co-precipitation is the prevailing technique for LDH production owing to the superior crystallinity and purity of the resulting LDH, as well as the straightforwardness of the approach (Abdallah et al. 2023). Additional techniques, such as hydrothermal, electrosynthesis, and sol–gel processes, have been documented in previous studies (Abdallah et al. 2023).

The co-precipitation approach entails adding a solution containing metal (II) and metal (III) cations in a certain ratio, resulting in the creation of hexa-aqua metal hybrids and brucite-like layers. The anion exchange approach involves the substitution of interlayer anions with desirable guest anions, using a suitable solvent and pH conditions. During the process of calcination, the anions located between the layers and the water molecules are eliminated. Rehydration or reconstruction takes place when the calcined-layered double hydroxide restores its structure after being exposed to liquids containing anions and water molecules. The approach enables precise manipulation of the composition and structure, considering the intricate composition and corresponding phases of LDHs (Jijoe et al. 2021).

LDHs have numerous advantageous characteristics that make them suitable for a wide range of applications. These properties encompass their capacity to exchange anions between intercalated and target anions, their high capability to eliminate anionic dyes through their reconstruction effect and rehydration when dispersed in anionic solutions (memory effect), and their extensive surface area, thermal stability, and the generation of more reactive species on the surface of photocatalysts when exposed to radiation (Janani et al. 2021). LDHs have been discovered to have advantageous uses as adsorbents and as precursors for metal oxide photocatalysts. Within the field of biomedicine, they function as promising carriers for delivering medications because of their chemical stability, capacity to coexist with living organisms, and ability to dissolve pharmaceuticals that have low solubility in a pH-dependent manner (Janani et al. 2021).

LDHs have been investigated as antibacterial agents against a wide range of microbes, in addition to their many uses. Dadakhani et al. (2024) conducted a recent and promising study in which they synthesized a histidine (His)-modified ZnCr LDH. The colorimetric approach was employed to evaluate the enzyme-like activity of His/ZnCr-LDH in this work. Later, a technique was developed that uses the strong peroxidase-like activity of His/ZnCr-LDH and its capacity to generate ROS in the presence of glucose oxidase (GOx) and glucose (Glu) as a source of hydrogen peroxide (H_2_O_2_). This approach had antibacterial properties boosted by acid. Gluconic acid (GA), the primary outcome of the glucose oxidase (GOx) process, creates an acidic milieu and enhances the production of ROS (Dadakhani et al. 2024). The results exhibited significant antibacterial efficacy at a low minimum inhibitory concentration (MIC), highlighting the potential of His/ZnCr-LDH for efficient suppression of microorganisms. The results of animal trials conducted by Dadakhani et al. (2024) showed that His/ZnCr-LDH successfully improved wound healing. This suggests that LDHs modified with amino acids present a new and effective approach for eliminating bacteria in different medicinal applications.

Tang et al. (2018) conducted research where they intercalated DL-mandelic acid (MA) into Zn-Al LDH using an anion exchange process. The antibacterial and antifungal properties of the ZnAl-MA-LDH compound were assessed using the agar well diffusion method against *Escherichia coli*, *Staphylococcus aureus*, and *Candida albicans* as representative examples of Gram-negative bacteria, Gram-positive bacteria, and fungi, respectively. The ZnAl LDH demonstrated a gradual and continuous release of mandelic anion at pH 4, suggesting its suitability for antibacterial activities in cosmetics. Furthermore, the enhanced and extended effectiveness of the new nanohybrid against *Escherichia coli*, *Staphylococcus aureus*, and *Candida albicans* indicates its potential use as an antibacterial substance in the pharmaceutical and cosmetic industries (Tang et al. 2018).

In addition, Tabti et al. (2020) produced a variety of Cu-LDHs by employing the co-precipitation approach. They utilized metal nitrates as precursors and sodium carbonate with different molar ratios of Cu/Al (Tabti et al. 2020). The antibacterial efficacy of Cu-LDHs with varying molar ratios of Cu/Al and their calcined phases was assessed against a range of bacteria, including *Escherichia coli*, *Pseudomonas aeruginosa*, *Enterococcus faecalis*, *Staphylococcus aureus*, and *Bacillus subtilis*. The study found that the Cu_0.10_–Al_0.10_-LDHs sample showed significant activity against all bacterial species. The calcined samples showed superior antibacterial activity compared to the non-calcined samples, regardless of the Cu/Al molar ratios (Tabti et al. 2020). The results indicated that Cu-LDHs have potential as antibacterial agents in the field of antibacterial research (Tabti et al. 2020). Other applications of LDH with antibacterial activities are summarized in Table S7.

## Limitations, challenges, and future perspectives

Despite the advantages and beneficial uses of these new materials, several challenges remain to be addressed. One significant concern is their potential toxicity; biosafety is crucial when designing antibacterial materials for clinical treatment. Issues such as the toxic effects on normal tissues and the in vivo degradation and excretion of these materials need thorough investigation. Current research primarily focuses on developing antibacterial agents and wound-healing dressings, but the poor dispersibility and long-lasting properties of pure compounds limit their broader applications in the antibacterial field. Additionally, the absorption, distribution, and metabolism of these compounds in various tissues require closer monitoring and further research (Guo et al. 2024).

Looking ahead, it is promising to design and develop biomacromolecule composites of these materials that exhibit strong binding force and enhance dispersibility for use in the surface disinfection of medical devices. Extensive research has shown that these new materials and their composites possess a wide antibacterial spectrum, high drug-loading capacity, and good biocompatibility. Future studies should aim to develop these materials and their composites with more stable and potent antibacterial activity for large-scale clinical applications (Guo et al. 2024). On the other hand, the preparation of these materials from waste and biomass is a promising area, not only from an economical point of view but also from a sustainability perspective (Mansour et al. 2024b; Hamed et al. 2024; Bedair et al. 2024e).

## Conclusion

Addressing the rising problem of antibiotic resistance necessitates the exploration and development of new materials with potent antibacterial properties. This review has highlighted the significant potential of various emerging materials, including MOFs, COFs, LDHs, QDs, and CQDs, along with ILs and DESs. These materials offer distinct advantages such as high surface area, porosity, and controlled release properties, making them promising candidates for diverse applications. Continued research and development in this field are essential to fully leverage the potential of these materials, paving the way for new, effective strategies to combat antibiotic resistance. The insights provided in this review underscore the importance of innovative materials in improving public health outcomes and addressing the global antibiotic resistance crisis.

## Supplementary Information

Below is the link to the electronic supplementary material.

Supplementary file1 (PDF 606 KB)

## Data Availability

Not applicable.
