# Supplementary material for: New emerging materials with potential antibacterial activities
Source: Appl Microbiol Biotechnol. 2024 Nov 14;108:515. doi: 10.1007/s00253-024-13337-6 (PMC11564324; doi:10.1007/s00253-024-13337-6)
Supplement: Supplementary file 1 [file 253_2024_13337_MOESM1_ESM.pdf]

New emerging materials with potential antibacterial activities

Hadeer M. Bedair[1], Mahmoud Hamed[2], Fotouh R. Mansour[3*]

[1] Department of Microbiology and Immunology, Faculty of Pharmacy, Misr University for Science and Technology, Egypt

[2] Pharmaceutical Chemistry Department, Faculty of Pharmacy, Misr International University, Km 28 Ismailia Road, Cairo 44971, Egypt

[3]Pharmaceutical Analytical Chemistry Department, Faculty of Pharmacy, Tanta University, Tanta, 31111, Egypt

Supplementary Material

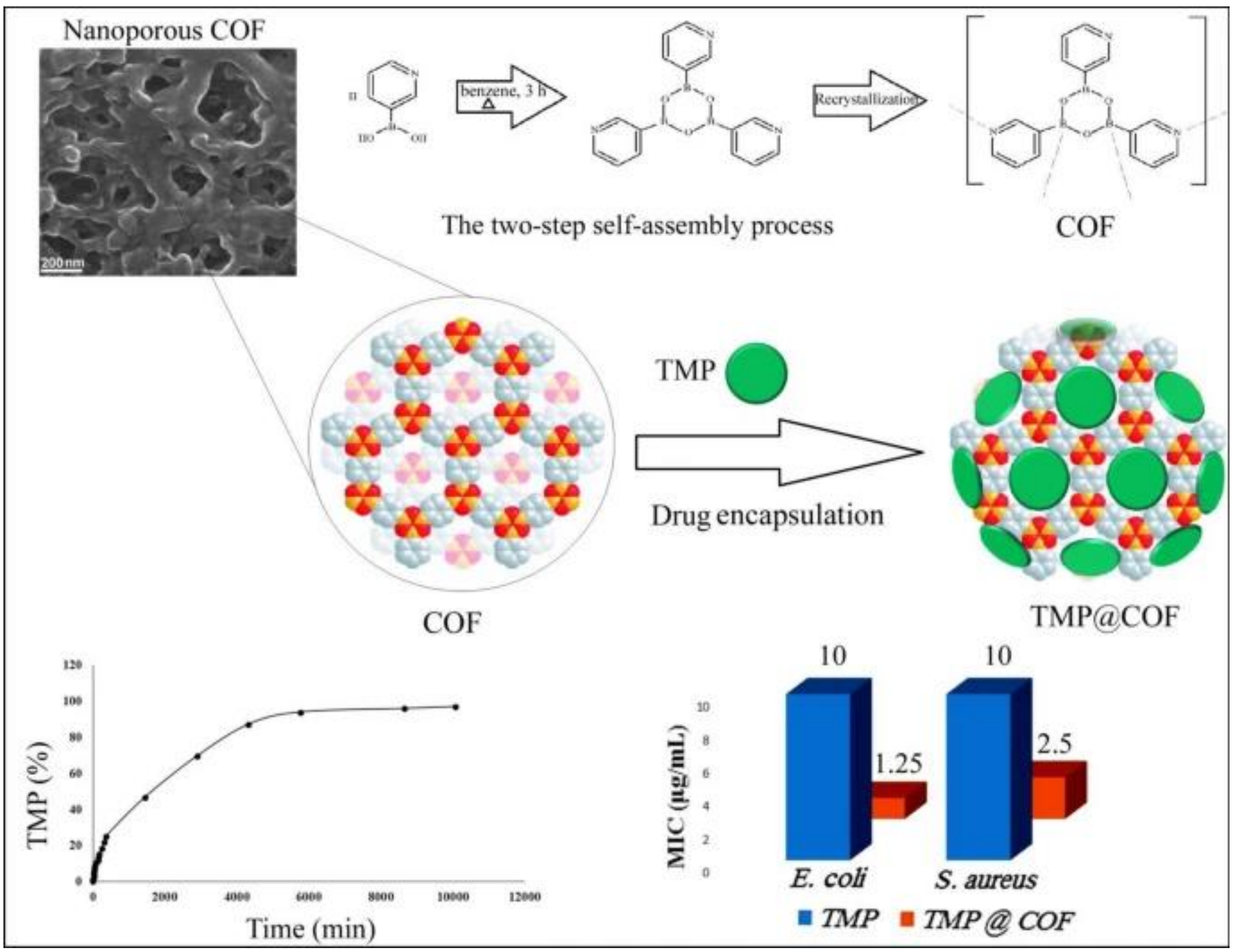

Figure S1: COFs as a delivery system for trimethoprim against *Escherichia coli* and *Staphylococcus aureus.* With permission from [1].

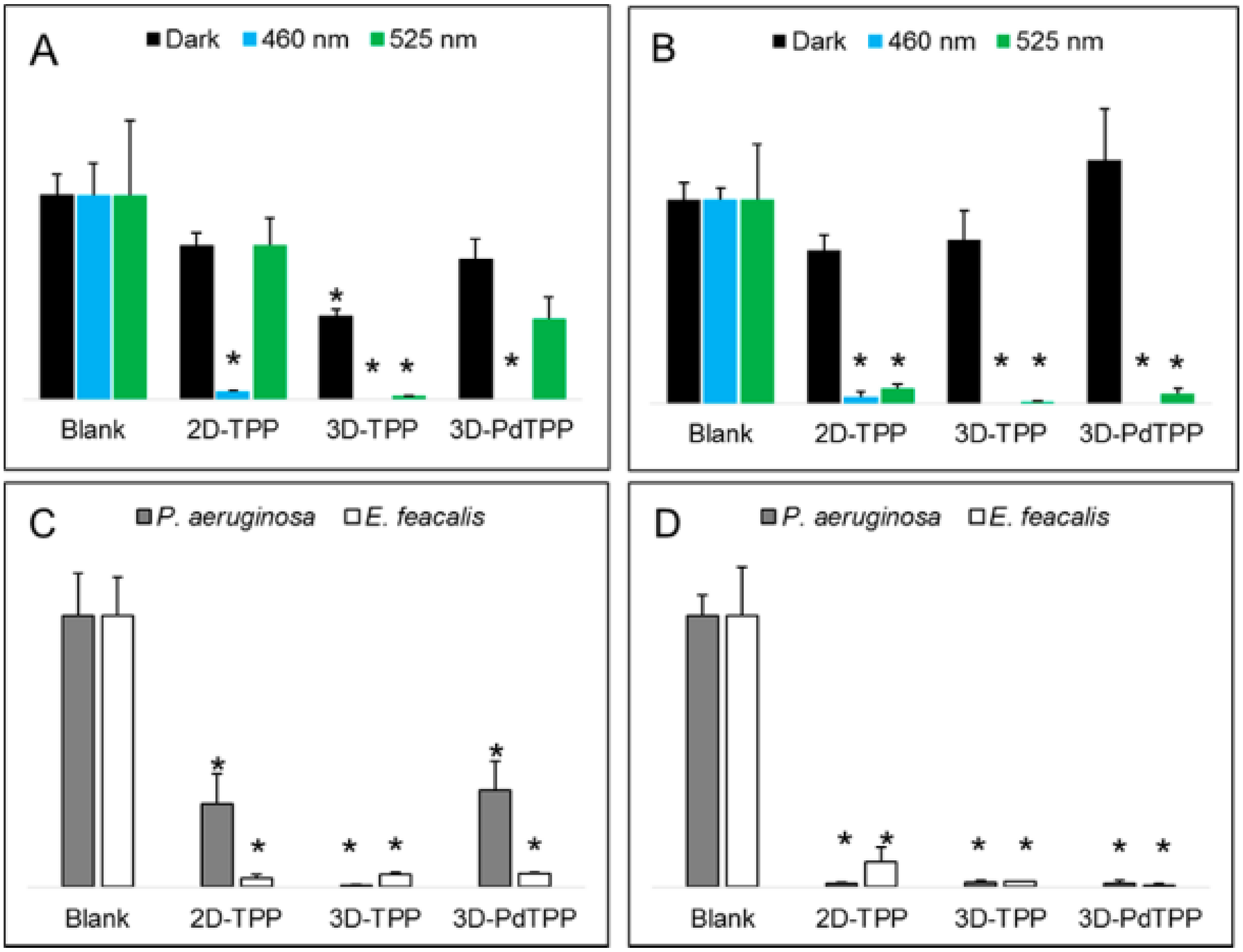

Figure S2: Antimicrobial activity of COF coatings. Surfaces were incubated for 24 hours in darkness (black) and under 460 nm (20 mW cm^{-2}, blue) or 525 nm (7 mW cm^{-2}, green) light exposure with *P. aeruginosa* (A) and *E. faecalis* (B). An additional experiment with 48 hours of incubation under 460 nm light was conducted (C). To assess direct biofilm cell killing, biofilms of *P. aeruginosa* and *E. faecalis* were grown with 24 hours of dark incubation followed by 4 hours of 460 nm light exposure (D). Control experiments used polymer coatings without COFs [2].

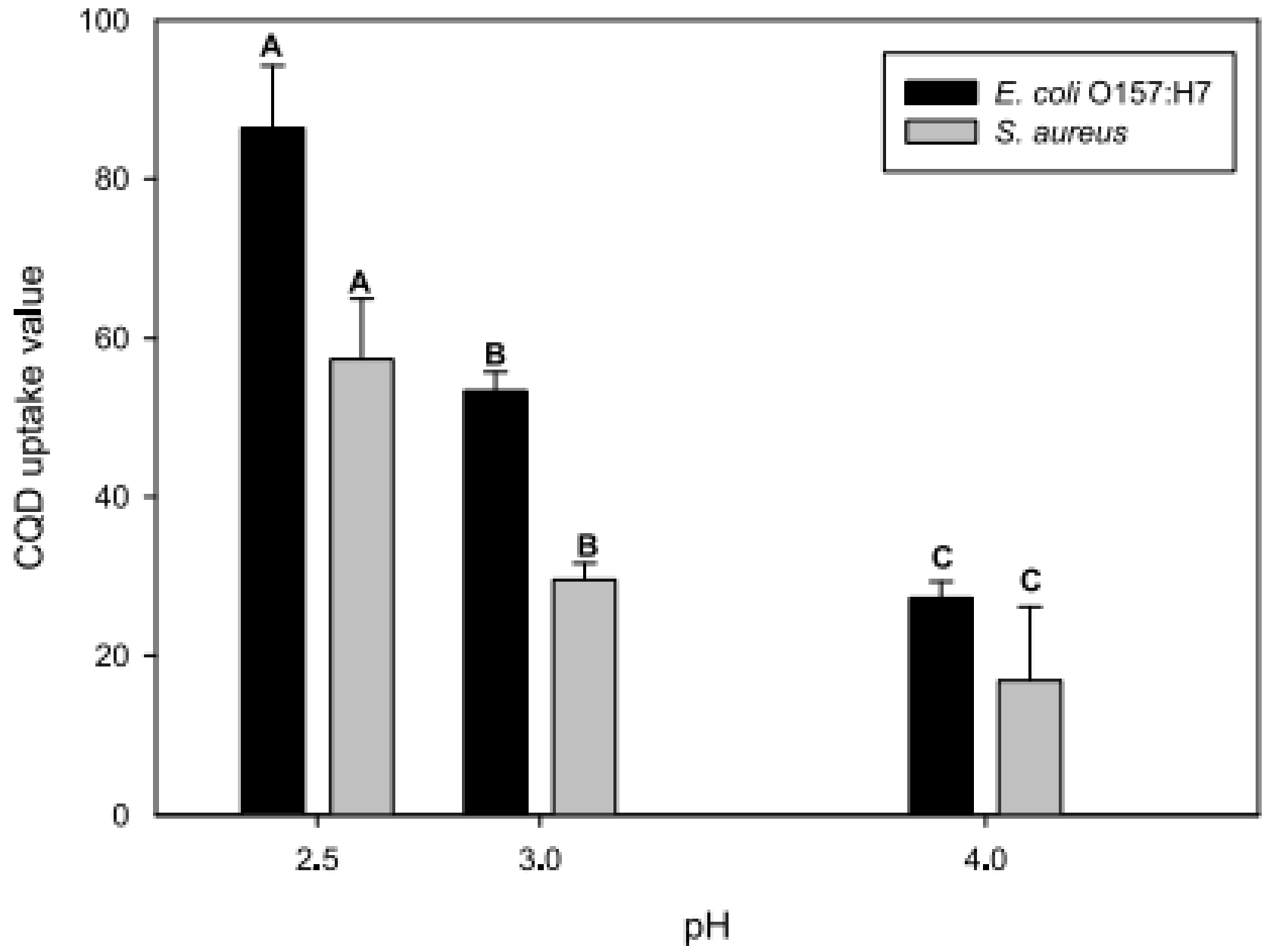

Figure S3: The carbon quantum dot (CQD) derived from spent coffee grounds (SCG) was assessed for its absorption capacity in *Staphylococcus aureus* and *Escherichia coli* O157:H7 cells under varying pH settings (2.5, 3.5, and 4.0). The data presented are the average of three separate studies, with the standard deviations indicated by the error bars. If the same capital letter is used within the same pathogen, it means that there is no statistically significant difference ($P > 0.05$). With the permission of [3].

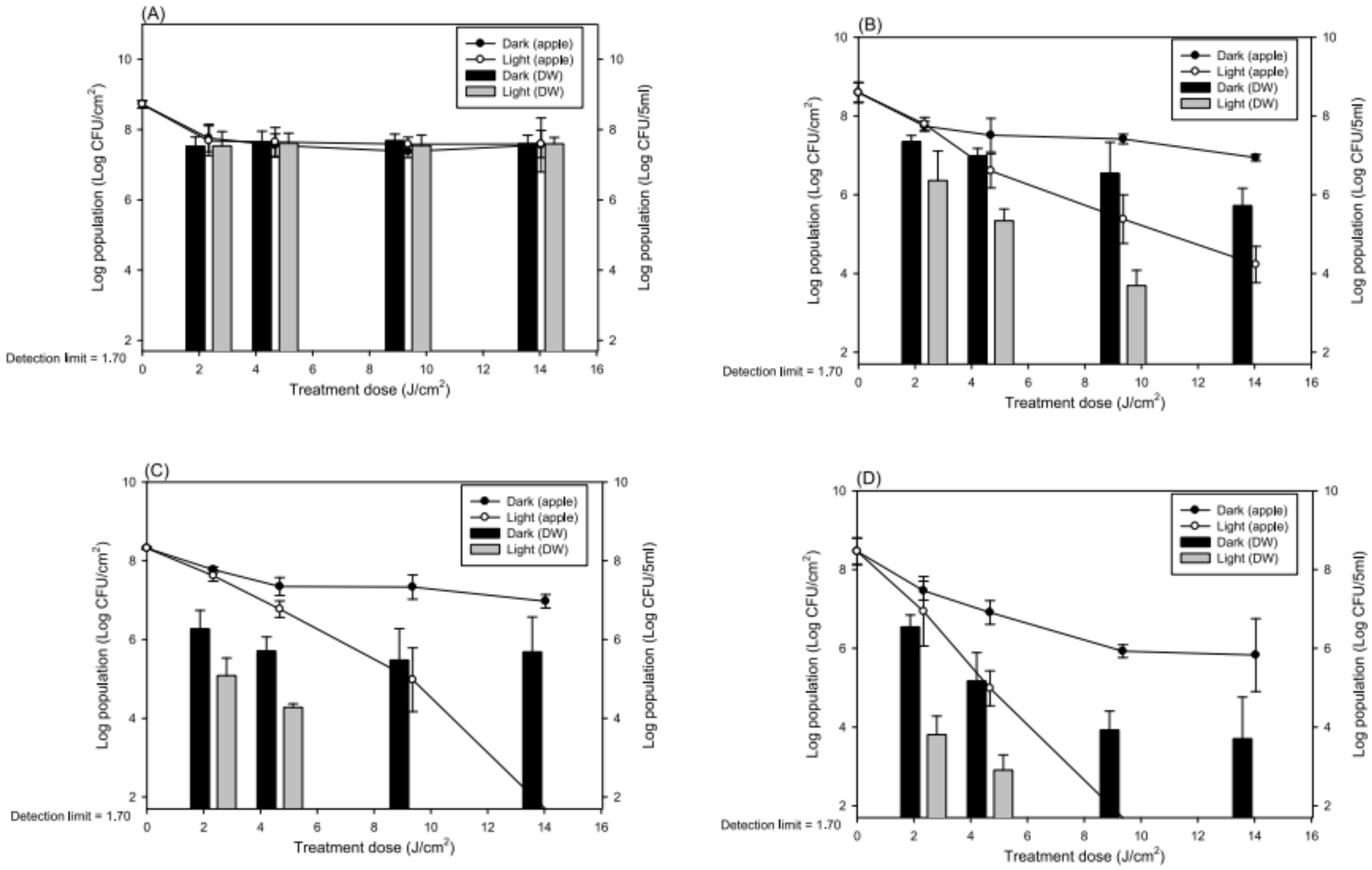

Figure S4: Measuring the survival rates of *Escherichia coli* O157:H7 on the surface of apples or in a washing solution (distilled water). Following treatment, a combination of carbon quantum dot produced from wasted coffee ground (at a concentration of 1 mg/ml) and varying concentrations of malic acid (A) 0%, B) 1.0%, C) 1.5%, and D) 2.0%) was applied. The treatment was conducted under both visible light irradiation and non-irradiation conditions. The data shown are the average of three separate studies, with the standard deviations indicated by error bars. With the permission of Kang et al 2024 [3].

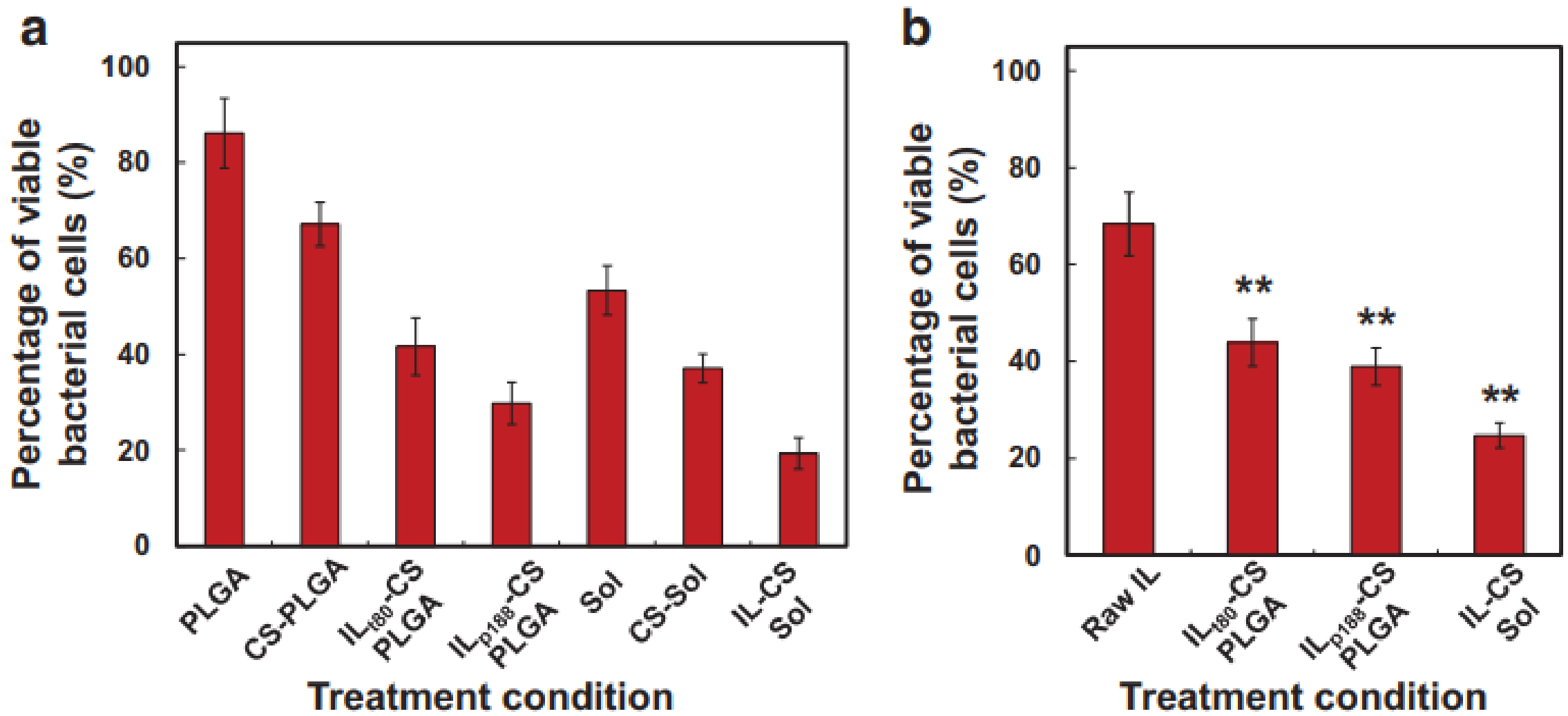

Figure S5: Percentage of viable bacterial cells following treatment with different types of nanoparticles (NPs) (NPs weight: 1 mg/well) (a) and different types of polymeric nanoparticles incorporating ionic liquids (IL concentration: 20 μg/well) (b). The data are presented as the mean value plus or minus the standard deviation, with a sample size of 3. There is a significant distinction between the untreated IL and other treatments, with a p-value of less than 0.01. With the permission of [4].

Table 1: Selected studies of MOFs as antibacterial

MOF/MOF-based system	MOF components	Method of preparation	Tested microorganism	Strain	Antibacterial assay: Plate/Colony counting approach	MIC/ Other techniques	Reference
PCN-224(Zr/Ti)	TCPP and $ZrCl_4$	Solvothermal	*Escherichia coli*	MDR	96.4% bacterial elimination	NR	[5]
PCN-224(Zr/Ti)	TCPP and $ZrCl_4$	Solvothermal	*Acinetobacter baumannii*	MDR	100% bacterial elimination	NR	[5]
PCN-224(Zr/Ti)	TCPP and $ZrCl_4$	Solvothermal	Methicillin-resistant *Staphylococcus epidermidis*	MRSE	96.2% bacterial elimination	NR	[5]
PCN-224(Zr/Ti)	TCPP and $ZrCl_4$	Solvothermal	Methicillin-resistant *Staphylococcus. aureus*	MRSA	96.8% bacterial elimination	NR	[5]

Cu-MOF	$Cu(NO_3)_2 \cdot 3H_2O$, glutaric acid and bpe	Hydrothermal	*Escherichia coli*	ATCC 25922	NR	MBC (20 µg/mL)	[6]
Cu-MOF	$Cu(NO_3)_2 \cdot 3H_2O$, glutaric acid and bpe	Hydrothermal	*Staphylococcus. aureus*	ATCC 6358	NR	MBC (20 µg/mL)	[6]
Zn-BTC	$Zn(NO_3)_2.6H_2O$ and H_3BTC	hydrothermal	Methicillin-resistant *Staphylococcus aureus*	MRSA	Antibacterial effect 41.4% colony plate assay	NR	[7]
Zn-BTC	$Zn(NO_3)_2.6H_2O$ and H_3BTC	hydrothermal	*Escherichia coli*	HB101(RP4)	Antibacterial effect 47.2% colony plate assay	NR	[7]

Table S2: Selected studies of COFs as antimicrobial agents

COF/COF system-used	COF/COF-system components	Method of preparation	Tested organism	Strain	Agar diffusion assay (ZOI) mm	Colony assay/plate counting	MIC	Reference
Ag/COF_{TGTp}	TG and Tp with AgNPs	Solvothermal	*Staphylococcus. aureus*	ATCC 25923	NR	At 100 μg/mL, bacterial inhibition 100%	50 μg/mL	[8]
Ag/COF_{TGTp}	TG and Tp with AgNPs	Solvothermal	*Escherichia coli*	ATCC 25922	NR	At 100 μg/mL, bacterial inhibition 100%	100 μg/mL	[8]
COFTDETA	Terephthaldehyde with diethylenetriamine	Solvothermal	*Escherichia coli*	NR	4	NR	6 mg/mL	[9]

COFTDETA	Terephthaldehyde with diethylenetriamine	Solvothermal	*Enterococcus faecalis*	NR	2	NR	8 mg/mL	[9]
COFTDETA	Terephthaldehyde with diethylenetriamine	Solvothermal	*Pseudomonas aeruginosa*	NR	5	NR	5 mg/mL	[9]
COFTDETA	Terephthaldehyde with diethylenetriamine	Solvothermal	*Staphylococcus. aureus*	NR	2	NR	6 mg/mL	[9]
COFs-AgNPs	AgNPs, TMC and PPD	Microwave irradiation (Zero-room temperature)	*Escherichia coli*	O157:H7	NR	At 60 µg/mL the total bacterial colony reduced by more than 90%.	60 µg/mL	[10]
COFs-AgNPs	AgNPs, TMC and PPD	Microwave irradiation	*Staphylococcus. aureus*	NR	NR	At 60 µg/mL the total bacterial colony	60 µg/mL	[10]

	reduced by more than 90%.	

Table S3: Selected studies of QDs as antibacterial agents

QDs used	Components	Method of preparation	Tested microorganism	Antibacterial assay	Reference
La-doped CeO2 QDs	$CeH_{12}N_3O_5$ and La $(NO_3)_3·6H_2O$	Hydrothermal	*Escherichia coli*	Agar diffusion ZOI 3.05 mm	[11]
ZAIS QDs@ZIF-8	Zn $(Ac)_2$, $AgNO_3$, In $(Ac)_3$, GSH , Zn $(NO_3)_2·6H_2O$ and 2-MeIM	Hydrothermal/ coordination-assisted self-assembly	*Escherichia coli*	cell density of bacteria decreased by 7.99 Log_{10}	[12]
ZAIS QDs@ZIF-8	Zn $(Ac)_2$, $AgNO_3$, In $(Ac)_3$, GSH , Zn $(NO_3)_2·6H_2O$ and 2-MeIM	Hydrothermal/ coordination-assisted self-assembly	*Staphylococcus aureus*	cell density of bacteria decreased by 5.23 Log_{10}	[12]
MoS_2 QDs	MoS_2 powder and N, N-dimethylformamide	Sonication and Solvothermal	*Escherichia coli*	At 50 µg/mL Survival rate 60% (Co-culture Assay)	[13]
MoS_2 QDs	MoS_2 powder and N, N-dimethylformamide	Sonication and Solvothermal	*Staphylococcus aureus*	At 50 µg/mL Survival rate 40% (Co-culture Assay)	[13]
ZnS QDs	$Zn(NO_3)_2·6H_2O$, Na_2S and D-Glucose	Modified (GCS) precipitation reaction	*Bacillus subtilis*	Agar diffusion (ZOI) 23 mm MIC/MBC (µg/ml) 75/125	[14]
ZnS QDs	$Zn(NO_3)_2·6H_2O$, Na_2S and D-Glucose	Modified (GCS) precipitation reaction	*Staphylococcus aureus*	Agar diffusion (ZOI) 29 mm MIC/MBC (µg/ml) 75/125	[14]

Type-II InP/ZnO core/shell QDs	$P(TMS)_3$, $InCl_3$, OA, OLA and ODE	Hot-injection method	*Pseudomonas aeruginosa* ATCC® 700829™	Plate count: at 50 µM 99.99% growth inhibition MIC (75-125 µM)	[15]
Type-II InP/ZnO core/shell QDs	$P(TMS)_3$, $InCl_3$, OA, OLA and ODE	Hot-injection method	*Escherichia coli*	Plate count: at 50 µM 61.81% growth inhibition	[15]

Table S4: Selected applications of CQDs as antibacterial agents

CQDs-used	Components	Method of preparation	Tested microorganism	Strain	Antibacterial assay	Reference
N-CQDs	Polyvinylpyrrolidone	Hydrothermal	*Bacillus subtilis*	ATCC 6051	Disk diffusion: 10 mm<br>MIC: 16 µg/mL	[16]
N-CQDs	Polyvinylpyrrolidone	Hydrothermal	*Escherichia coli*	CECT 831	Disk diffusion: 9.8 mm<br>MIC: 32 µg/mL	[16]
PC-CQDs	CA, GSH, PEPA and Acetone	Solvothermal	*Escherichia coli*	NR	MIC: 120 µg/mL<br>Disc diffusion: 15.01mm	[17]
PC-CQDs	CA, GSH, PEPA and Acetone	Solvothermal	Methicillin-resistant *Staphylococcus aureus*	NR	MIC:30 µg/mL<br>Disc diffusion:12.27 mm	[17]
PC-CQDs	CA, GSH, PEPA and Acetone	Solvothermal	*Pseudomonas aeruginosa*	NR	MIC:120 µg/mL	[17]
PC-CQDs	CA, GSH, PEPA and Acetone	Solvothermal	*Enterococcus faecalis*	NR	MIC:60 µg/mL	[17]
PC-CQDs	CA, GSH, PEPA and Acetone	Solvothermal	Drug-resistant *Pseudomonas aeruginosa*	Clinical	MIC:480 µg/mL	[17]
PC-CQDs	CA, GSH, PEPA and Acetone	Solvothermal	Drug-resistant *Escherichia coli*	Clinical	MIC:480 µg/mL	[17]
PC-CQDs	CA, GSH, PEPA and Acetone	Solvothermal	*Listeria. monocytogenes*	NR	MIC:30 µg/mL	[17]
PC-CQDs	CA, GSH, PEPA and Acetone	Solvothermal	*Serratia.marcescens*	NR	MIC:240 µg/mL	[17]

PC-CQDs	CA, GSH, PEPA and Acetone	Solvothermal	*Staphylococcus aureus*	NR	MIC:15 µg/mL<br>Disc diffusion: 15.24 mm	[17]
NCQDs	Glucose, DETA, ethanol, chloral hydrate, GSH and GSSG	Hydrothermal	*Staphylococcus aureus*	ATCC 6538	Disc diffusion: 14.5 mm<br>MIC: 0.256 mg/mL	[18]
NCQDs	Glucose, DETA, ethanol, chloral hydrate, GSH and GSSG	Hydrothermal	*Staphylococcus aureus*	ATCC 43300	Disc diffusion :15.5 mm	[18]
NCQDs	Glucose, DETA, ethanol, chloral hydrate, GSH and GSSG	Hydrothermal	*Staphylococcus epidermidis*	ATCC 12228	Disc diffusion :14.5mm	[18]
NCQDs	Glucose, DETA, ethanol, chloral hydrate, GSH and GSSG	Hydrothermal	Methicillin-resistant *Staphylococcus aureus*	NR	Disc diffusion:14.5mm<br>MIC:0.128 mg/mL	[18]
qCQDs	DDA, glucose	Solvothermal	*Staphylococcus* epidermidis	ATCC 12228	Disc diffusion: 14mm<br>MIC: 13 µg/mL	[19]

Table S5: Selected studies illustrating the use of ILs as antibacterial agents

ILs/ILs-Hybrid	Components	Method of preparation	Tested microorganism	Strain	Antibacterial assay	Reference
CABILs: [Ch][Lys] [Ch][Arg] [Ch][His]	loofah fiber, epoxy resin and CABILs	Heating and stirring	*Escherichia coli*	ATCC25922	No inhibition zone	[20]
CABILs: [Ch][Lys] [Ch][Arg] [Ch][His]	loofah fiber, epoxy resin and CABILs	Heating and stirring	*Staphylococcus aureus*	ATCC6538	Bacteriostatic rate L: 62.017% SL:23.692% SA:81.198% SH:0.379%	[20]
CnMPBr	1-methylpyrrolidine, ethyl acetate, CnH_2n+1Br and PVA	Stirring	*Escherichia coli*	CGMCC 1.12883	Agar diffusion S1	[21]
			Staphylococcus aureus	CMCC 26003	Agar diffusion S1	[21]
$[C_2OHMIM]$[seco-Amx]	C_2OHMIM ,Methanol, amoxicillin, ammonium solution, and methanol/acetonitrile		*Staphylococcus aureus*	ATCC 25923	Broth micro dilution: MIC 0.05 mM	[22]

[C_2OHMIM][seco-Amx]	C_2OHMIM ,Methanol, amoxicillin, ammonium solution, and methanol/acetonitrile	*Escherichia coli*	ATCC 25922	Broth micro dilution: MIC 5 mM	[22]
[C_2OHMIM][seco-Amx]	C_2OHMIM ,Methanol, amoxicillin, ammonium solution, and methanol/acetonitrile	Methicillin resistant *Staphylococcus aureus*	ATCC 43300	Broth micro dilution: MIC 5 mM	[22]
[C_2OHMIM][seco-Amx]	C_2OHMIM ,Methanol, amoxicillin, ammonium solution, and methanol/acetonitrile	*Escherichia coli*	CTX M2 and	Broth micro dilution: MIC >5 mM	[22]
[C_2OHMIM][seco-Amx]	C_2OHMIM ,Methanol, amoxicillin, ammonium solution, and methanol/acetonitrile	*Escherichia coli*	CTX M9	Broth micro dilution: MIC >5 mM	[22]

Table S6: Selected studies on using DES as antimicrobial agents

DES used	HBD	HBA	Ratio(HBD/HBA)	Method of preparation	Tested microorganism	Strain	Antibacterial assay	Reference
CA:LA	Lauric acid	Capric acid	1:2	Heating and stirring	*Staphylococcus aureus*	ATCC 25923	Disk diffusion: 15.67 ± 0.58 mm<br>MIC: 625 µg/mL	[23]
CA:LA	Lauric acid	Capric acid	1:2	Heating and stirring	*Candida albicans*	ATCC 90029	Disk diffusion: 13.5 ± 0.41 mm<br>MIC: 625 µg/mL	[23]
CA:LA	Lauric acid	Capric acid	1:2	Heating and stirring	*Escherichia coli*	ATCC 25922	Disk diffusion: No inhibition	[23]
CA:LA	Lauric acid	Capric acid	1:2	Heating and stirring	*Pseudomonas aeruginosa*	ATCC 27853	Disk diffusion: No inhibition	[23]
CA:LA	Lauric acid	Capric acid	1:2	Heating and stirring	Methicillin-resistant *Staphylococcus aureus*	ATCC 700698	Disk diffusion: 16.50 ± 0.41 mm<br>MIC: 625 µg/mL	[23]

CA:LA	Lauric acid	Capric acid	1:2	Heating and stirring	Methicillin-resistant *Staphylococcus. epidermis*	ATCC 35984	Disk diffusion: 20 ± 0.82mm MIC: 625 µg/mL	[23]
ChCl:GLY	Glycerol	Choline chloride	2:1	Thermal mixing	*Arthrobacter simplex*	TCCC 11037	Disk diffusion:19.8 ± 4.1	[24]
ChCl: 1,2-Propanediol	1,2-Propanediol	Choline chloride	2:1	Heating and stirring	*Clostridium perfringens*	ATCC 13124	Disk diffusion: (13-17) mm	[25]
ChCl: Oxalic acid:EG	Oxalic acid/EG	Choline chloride	1:1:1	Heating and stirring	*Escherichia coli*	ATCC 23564	Disk diffusion: 29 mm	[26]
Cit:Fru:Gly	Fructose/glycerol	Citric acid	1:1:1	Heating and stirring	*Escherichia coli*	3014	Disk diffusion: 50 ± 4	[27]
Cit:Fru:Gly	Fructose/glycerol	Citric acid	1:1:1	Heating and stirring	*Proteus. mirabilis*	3008	Disk diffusion: 81 ± 2 mm	[27]
Cit:Fru:Gly	Fructose/glycerol	Citric acid	1:1:1	Heating and stirring	*Salmonella. typhimurium*	3064	Disk diffusion: 55 ± 1 mm	[27]
Cit:Fru:Gly	Fructose/glycerol	Citric acid	1:1:1	Heating and stirring	*Pseudomonas. aeruginosa*	3024	Disk diffusion: 51 ± 4 mm	[27]
Cit:Fru:Gly	Fructose/glycerol	Citric acid	1:1:1	Heating and stirring	*Staphylococcus. aureus*	3048	Disk diffusion: 51 ± 3 mm	[27]

Cit:Fru:Gly	Fructose/glycerol	Citric acid	1:1:1	Heating and stirring	*Candida. albicans*	86	Disk diffusion: no inhibition	[27]
Be:Ma	Malic acid	Betaine	1:1	Ultrasonic irradiation	*Escherichia coli*	NR	Disk diffusion: 7.12 mm	[28]
Methyl-trioctylammonium chloride-based DES	Glycerol	Methyl-trioctylammonium chloride	1:1	Heating and stirring	*Escherichia coli K1*	MTCC 710859	Plate count: antibacterial 68%	[29]
Methyl-trioctylammonium chloride-based DES	Glycerol	Methyl-trioctylammonium chloride	1:1	Heating and stirring	*Pseudomonas aeruginosa*	ATCC 10145	Plate count: antibacterial 40%	[29]
Methyl-trioctylammonium chloride-based DES	Glycerol	Methyl-trioctylammonium chloride	1:1	Heating and stirring	*Streptococcus pneumoniae*	ATCC 33400	Plate count: antibacterial 60%	[29]
Methyl-trioctylammonium chloride-based DES	Glycerol	Methyl-trioctylammonium chloride	1:1	Heating and stirring	*Streptococcus pyogenes*	ATCC 12344	Plate count: antibacterial 50%	[29]

ChCl:G	Glycerol	Choline chloride	1:1	The vacuum evaporation	*Staphylococcus aureus*	ATCC 6538	MIC : 25%	[30]
ChCl:G	Glycerol	Choline chloride	1:1	The vacuum evaporation	*Pseudomonas aeruginosa*	ATCC 9027	MIC: 20%	[30]

Table S7: Selected studies on LDH as antimicrobial agents

LDH used	Method of preparation	Tested microorganism	Strain	Antibacterial assay	Reference
Zn-Al-MA-LDH	Ion-exchange	*Escherichia coli*	ATCC8739	Agar well diffusion: 15.64 mm	[31]
Zn-Al-MA-LDH	Ion-exchange	*Staphylococcus aureus*	ATCC6538	Agar well diffusion: 18,52 mm	[31]
Zn-Al-MA-LDH	Ion-exchange	*Candida albicans*	ATCC 10231	Agar well diffusion: 11.53 mm	[31]
Ni-La-LDO/ Fe_3O_4	Co-precipitation	*Escherichia coli*	ATCC® 11775™	Plate counting: 10^4 CFU/mL inactivated after 2 hr	[32]
Zn/Al-LDH-GA-loaded CMC films	Co-precipitation	*Escherichia coli*	ATCC 25922	Disk diffusion:30 mm, viable cell count: 20% RP	[33]
Zn/Al-LDH-GA-loaded CMC films	Co-precipitation	*Staphylococcus aureus*	ATCC 25923	Disk diffusion:25 mm, viable cell count:99% RP	[33]
His/ZnCr-LDH	Co-precipitation	*Staphylococcus aureus*	NR	Disk diffusion: 30 mm MIC: 3.5 µg/ mL	[34]
His/ZnCr-LDH	Co-precipitation	*Escherichia coli*	NR	Disk diffusion: 20 mm MIC:6 µg/ mL	[34]

References

[1] A. Salehi, M. Behpour, D. Afzali, Investigation into the antibacterial activity of covalent organic frameworks as a delivery system of trimethoprim against Escherichia coli and Staphylococcus aureus, Polym. Bull. 80 (2023) 1447–1461. doi:10.1007/s00289-022-04119-z.

[2] J. Hynek, J. Zelenka, J. Rathouský, P. Kubát, T. Ruml, J. Demel, et al., Designing Porphyrinic Covalent Organic Frameworks for the Photodynamic Inactivation of Bacteria, ACS Appl. Mater. Interfaces. 10 (2018) 8527–8535. doi:10.1021/acsami.7b19835.

[3] J.W. Kang, J.Y. Kim, D.H. Kang, Synthesis of carbon quantum dot synthesized using spent coffee ground as a biomass exhibiting visible-light-driven antimicrobial activity against foodborne pathogens, J. Food Eng. 365 (2024) 111820. doi:10.1016/j.jfoodeng.2023.111820.

[4] C. Takahashi, Y. Hattori, S. Yagi, T. Murai, M. Tanemura, Y. Kawashima, et al., Ionic liquid-incorporated polymeric nanoparticles as carriers for prevention and at an earlier stage of periodontal disease, Materialia. 8 (2019). doi:10.1016/j.mtla.2019.100395.

[5] M. Chen, Z. Long, R. Dong, L. Wang, J. Zhang, S. Li, et al., Titanium Incorporation into Zr-Porphyrinic Metal–Organic Frameworks with Enhanced Antibacterial Activity against Multidrug-Resistant Pathogens, Small. 16 (2020) 1–11. doi:10.1002/smll.201906240.

[6] K. Gwon, I. Han, S. Lee, Y. Kim, D.N. Lee, Novel Metal-Organic Framework-Based Photocrosslinked Hydrogel System for Efficient Antibacterial Applications, ACS Appl. Mater. Interfaces. 12 (2020) 20234–20242. doi:10.1021/acsami.0c03187.

[7] Y. Chen, J. Cai, D. Liu, S. Liu, D. Lei, L. Zheng, et al., Zinc-based metal organic framework with antibacterial and anti-inflammatory properties for promoting wound healing, Regen. Biomater. 9 (2022). doi:10.1093/rb/rbac019.

[8] H. Zhang, J. Ma, C. Liu, L. Li, C. Xu, Y. Li, et al., Antibacterial activity of guanidinium-based ionic covalent organic framework anchoring Ag nanoparticles, J. Hazard. Mater. 435 (2022) 128965. doi:10.1016/j.jhazmat.2022.128965.

[9] E.A. Gendy, A.I. Khodair, A.M. Fahim, D.T. Oyekunle, Z. Chen, Synthesis, characterization, antibacterial activities, molecular docking, and computational investigation of novel imine-linked covalent organic framework, J. Mol. Liq. 358 (2022) 119191. doi:10.1016/j.molliq.2022.119191.

[10] X. Dai, S. Li, S. Li, K. Ke, J. Pang, C. Wu, et al., High antibacterial activity of chitosan films with covalent organic frameworks immobilized silver nanoparticles, Int. J. Biol. Macromol. 202 (2022) 407–417. doi:10.1016/j.ijbiomac.2021.12.174.

[11] A. Shahzadi, S. Moeen, A.D. Khan, A. Haider, J. Haider, A. Ul-Hamid, et al., La-Doped CeO2 Quantum Dots: Novel Dye Degrader, Antibacterial Activity, and In Silico Molecular Docking Analysis, ACS Omega. 8 (2023) 8605–8616. doi:10.1021/acsomega.2c07753.

[12] M. Wang, L. Nian, Y. Cheng, B. Yuan, S. Cheng, C. Cao, Encapsulation of colloidal semiconductor quantum dots into metal-organic frameworks for enhanced antibacterial activity through interfacial electron transfer, Chem. Eng. J. 426 (2021) 130832. doi:10.1016/j.cej.2021.130832.

[13] X. Tian, Y. Sun, S. Fan, M.D. Boudreau, C. Chen, C. Ge, et al., Photogenerated Charge Carriers in Molybdenum Disulfide Quantum Dots with Enhanced Antibacterial Activity, ACS Appl. Mater. Interfaces. 11 (2019) 4858–4866. doi:10.1021/acsami.8b19958.

[14] J. Maheswari, B. Sanjeeb, K. Jyoti, Green chemistry synthesis of biocompatible ZnS quantum dots (QDs): their application as potential thin films and antibacterial agent, Int. Nano Lett. 9 (2019) 149–159. doi:10.1007/s40089-019-0270-x.

[15] S.U. Khan, G.O. Eren, N. Atac, A. Onal, M.H. Qureshi, F.K. Cooper, et al., Antibacterial type-II InP/ZnO quantum dots via multimodal reactive oxygen species, Chem. Eng. J. 480 (2024) 148140. doi:10.1016/j.cej.2023.148140.

[16] N.A. Travlou, D.A. Giannakoudakis, M. Algarra, A.M. Labella, E. Rodríguez-Castellón, T.J. Bandosz, S- and N-doped carbon quantum dots: Surface chemistry dependent antibacterial activity, Carbon N. Y. 135 (2018) 104–111. doi:10.1016/j.carbon.2018.04.018.

[17] X. Hao, L. Huang, C. Zhao, S. Chen, W. Lin, Y. Lin, et al., Antibacterial activity of

positively charged carbon quantum dots without detectable resistance for wound healing with mixed bacteria infection, Mater. Sci. Eng. C. 123 (2021) 111971. doi:10.1016/j.msec.2021.111971.

[18] C. Zhao, X. Wang, L. Wu, W. Wu, Y. Zheng, L. Lin, et al., Nitrogen-doped carbon quantum dots as an antimicrobial agent against Staphylococcus for the treatment of infected wounds, Colloids Surfaces B Biointerfaces. 179 (2019) 17–27. doi:10.1016/j.colsurfb.2019.03.042.

[19] C. Zhao, X. Wang, L. Yu, L. Wu, X. Hao, Q. Liu, et al., Acta Biomaterialia Quaternized carbon quantum dots with broad-spectrum antibacterial activity for the treatment of wounds infected with mixed bacteria, Acta Biomater. 138 (2022) 528–544. doi:10.1016/j.actbio.2021.11.010.

[20] X. Gao, Q. He, G. Zhou, A. Ali, C. Yu, S. Yao, Choline and amino acids-based ionic liquids (CABILs) for the preparation of new antibacterial coating with loofah and epoxy resin, Ind. Crops Prod. 210 (2024) 118093. doi:10.1016/j.indcrop.2024.118093.

[21] Y. Yu, Z. Yang, S. Ren, Y. Gao, L. Zheng, Multifunctional hydrogel based on ionic liquid with antibacterial performance, J. Mol. Liq. 299 (2020) 112185. doi:10.1016/j.molliq.2019.112185.

[22] R. Ferraz, D. Silva, A.R. Dias, V. Dias, M.M. Santos, L. Pinheiro, et al., Synthesis and antibacterial activity of ionic liquids and organic salts based on penicillin g and amoxicillin hydrolysate derivatives against resistant bacteria, Pharmaceutics. 12 (2020). doi:10.3390/pharmaceutics12030221.

[23] J.M. Silva, E. Silva, R.L. Reis, A.R.C. Duarte, A closer look in the antimicrobial properties of deep eutectic solvents based on fatty acids, Sustain. Chem. Pharm. 14 (2019) 100192. doi:10.1016/j.scp.2019.100192.

[24] S. Mao, K. Li, Y. Hou, Y. Liu, S. Ji, H. Qin, et al., Synergistic effects of components in deep eutectic solvents relieve toxicity and improve the performance of steroid biotransformation catalyzed by Arthrobacter simplex, J. Chem. Technol. Biotechnol. 93 (2018) 2729–2736. doi:10.1002/jctb.5629.

[25] J.P. Wojeicchowski, C. Marques, L. Igarashi-Mafra, J.A.P. Coutinho, M.R. Mafra, Extraction of phenolic compounds from rosemary using choline chloride – based Deep

Eutectic Solvents, Sep. Purif. Technol. 258 (2021). doi:10.1016/j.seppur.2020.117975.

[26] A.K. Jangir, B. Lad, U. Dani, N. Shah, K. Kuperkar, In vitro toxicity assessment and enhanced drug solubility profile of green deep eutectic solvent derivatives (DESDs) combined with theoretical validation, RSC Adv. 10 (2020) 24063–24072. doi:10.1039/c9ra10320a.

[27] K. Radošević, I. Čanak, M. Panić, K. Markov, M.C. Bubalo, J. Frece, et al., Antimicrobial, cytotoxic and antioxidative evaluation of natural deep eutectic solvents, Environ. Sci. Pollut. Res. 25 (2018) 14188–14196. doi:10.1007/s11356-018-1669-z.

[28] Y. Liang, Z. Pan, Z. Chen, Y. Fei, J. Zhang, J. Yuan, et al., Ultrasound-Assisted Natural Deep Eutectic Solvents as Separation-Free Extraction Media for Hydroxytyrosol from Olives, ChemistrySelect. 5 (2020) 10939–10944. doi:10.1002/slct.202002026.

[29] N. Akbar, N.A. Khan, T. Ibrahim, M. Khamis, A.S. Khan, A.M. Alharbi, et al., Antimicrobial Activity of Novel Deep Eutectic Solvents, Sci. Pharm. 91 (2023). doi:10.3390/scipharm91010009.

[30] H.L. Nystedt, K.G. Grønlien, R.R. Rolfsnes, H.C. Winther-Larsen, O.A. Løchen Økstad, H.H. Tønnesen, Neutral natural deep eutectic solvents as anti-biofilm agents, Biofilm. 5 (2023). doi:10.1016/j.bioflm.2023.100114.

[31] L.P. Tang, H.M. Cheng, S.M. Cui, X.R. Wang, L.Y. Song, W. Zhou, et al., DL-mandelic acid intercalated Zn-Al layered double hydroxide: A novel antimicrobial layered material, Colloids Surfaces B Biointerfaces. 165 (2018) 111–117. doi:10.1016/j.colsurfb.2018.02.017.

[32] C.T. Vu, T. Wu, Magnetic porous NiLa-Layered double oxides (LDOs) with improved phosphate adsorption and antibacterial activity for treatment of secondary effluent, Water Res. 175 (2020) 115679. doi:10.1016/j.watres.2020.115679.

[33] S. Barkhordari, A. Alizadeh, Zinc/aluminum-layered double hydroxide-gallic acid doped carboxymethyl cellulose nanocomposite films for wound healing, Int. J. Biol. Macromol. 260 (2024) 129556. doi:10.1016/j.ijbiomac.2024.129556.

[34] S. Dadakhani, G. Dehghan, A. Khataee, A. Erfanparast, Design and application of histidine-functionalized ZnCr-LDH nanozyme for promoting bacteria-infected wound

healing, RSC Adv. 14 (2024) 1195–1206. doi:10.1039/d3ra07364e.
